# Mortality trends in the co-occurrence of urinary tract cancer and diabetes mellitus

**DOI:** 10.3389/fendo.2026.1843044

**Published:** 2026-05-22

**Authors:** Chen-Zhang Ou, Yu-Jun Xiong, Xiang-Da Meng, Tian Lv, Lide Song

**Affiliations:** 1 Department of Urology, Wuxi No.2 People’s Hospital, Jiangnan University Medical Center, Wuxi, Jiangsu, China; 2 Department of Gastroenterology, Beijing Hospital, National Center for Gerontology, National Clinical Research Center for Gerontology, The Key Laboratory of Geriatrics of NHC, Institute of Geriatric Medicine, Chinese Academy of Medical Sciences, Beijing, China; 3 Department of Hernia and Abdominal Wall Surgery, Peking University People’s Hospital, Beijing, China; 4 Department of Neurology, Zhuji Affiliated Hospital of Wenzhou Medical University, Zhuji, China; 5 Department of Urology, Zhuji Affiliated Hospital of Wenzhou Medical University, Zhuji, China

**Keywords:** age-adjusted mortality rate, APC, diabetes, mortality trends, urinary tract cancer

## Abstract

**Background:**

Urinary tract cancers and diabetes mellitus often co−occur and may interact to worsen clinical outcomes, yet long−term mortality patterns in this population remain poorly understood.

**Methods:**

Using national death certificate data from 1999 to 2024, we identified adults aged ≥25 years with both urinary tract cancers (ICD−10: C64–C68) and diabetes mellitus (ICD−10: E10–E14). Age−adjusted mortality rates (AAMRs) were standardized to the 2000 U.S. population. Temporal trends were assessed via Joinpoint regression to estimate annual percent changes (APC) and average annual percent changes (AAPC). Analyses were stratified by age, sex, race, region, and urbanization level.

**Results:**

Deaths increased from 1, 566 in 1999 to 4, 487 in 2024; the AAMR rose from 0.87 to 1.53 per 100, 000 (AAPC 2.30%; 95% CI: 1.32%–3.29%). Mortality surged during 2018–2021 (APC 9.93%) before leveling off. Men had higher rates and a steeper rise than women (AAPC 2.42% vs. 1.00%). The oldest age group (≥85 years) showed the largest increase (AAPC 3.61%). Geographically, the South outpaced other regions (AAPC 3.57%), and nonmetropolitan areas exceeded metropolitan ones in both mortality level and growth (AAPC 3.19% vs. 1.85%). Among racial groups, non−Hispanic White individuals experienced the greatest rise (AAPC 2.51%).

**Conclusions:**

Over two decades, mortality from coexisting urinary tract cancers and diabetes rose substantially, with marked demographic and geographic disparities. Integrated prevention and management strategies are urgently needed.

## 1 Introduction

Urinary tract cancers, encompassing malignancies of the bladder, ureter, and renal pelvis, represent a significant global health burden, with substantial morbidity and mortality across diverse populations (1). Concurrently, diabetes mellitus has emerged as a prevalent metabolic disorder worldwide, characterized by chronic hyperglycemia and systemic complications affecting multiple organ systems (2). Increasing evidence suggests that diabetes frequently coexists with urinary tract cancers and may adversely influence cancer development, progression, and therapeutic outcomes (3). Among patients with malignancies, the prevalence of diabetes is notably high, reflecting shared risk factors such as advanced age, obesity, and lifestyle-related exposures.

Several epidemiological and clinical studies have demonstrated that diabetes is associated with an elevated risk of developing urinary tract cancers, particularly bladder cancer (4). Potential mechanisms underlying this association include hyperinsulinemia, insulin resistance, and chronic low-grade inflammation, which may promote carcinogenesis through activation of proliferative and anti-apoptotic signaling pathways (5). In addition, oxidative stress and the accumulation of advanced glycation end products in diabetic individuals may contribute to DNA damage and tumor initiation (6). Beyond its role in cancer risk, diabetes has also been linked to worse oncologic outcomes, including increased recurrence rates, reduced response to therapy, and higher cancer-specific and all-cause mortality (7).

The coexistence of diabetes in patients with urinary tract cancers presents important clinical challenges. Diabetes-related comorbidities, such as cardiovascular disease and renal impairment, may limit treatment options and increase the risk of perioperative and treatment-related complications (8, 9). Moreover, certain antidiabetic medications and cancer therapies may interact, further complicating disease management. Despite growing recognition of these interactions, the population-level burden and temporal patterns of mortality among individuals with concurrent urinary tract cancers and diabetes remain insufficiently characterized.

In addition, the COVID-19 pandemic may have further amplified the burden of coexisting urinary tract cancers and diabetes mellitus. Widespread disruptions in healthcare delivery, including delays in cancer screening, diagnosis, treatment, and routine diabetes care, have been reported since 2020 (10). Individuals with diabetes and malignancy are also more vulnerable to severe infection and adverse outcomes following SARS-CoV-2 exposure. These factors may have contributed to recent fluctuations in mortality trends and underscore the importance of evaluating long-term population-level patterns in this clinically vulnerable group.

To address this gap, a retrospective observational study was conducted to evaluate long-term mortality trends among individuals with coexisting urinary tract cancers and diabetes over the past two decades. Mortality patterns were examined across demographic subgroups, including age, sex, and racial background, as well as geographic variation. By elucidating these epidemiological trends, the present study aims to provide a comprehensive understanding of the evolving burden of this comorbidity and to inform targeted strategies for improving clinical management and reducing disparities in outcomes.

## 2 Materials and methods

### 2.1 Study design and inclusion criteria

Mortality data were obtained from the U.S. Centers for Disease Control and Prevention Wide-ranging Online Data for Epidemiologic Research (CDC WONDER) database, a publicly accessible platform providing national death certificate data from the National Vital Statistics System (NVSS). Causes of death were classified according to the International Classification of Diseases, Tenth Revision (ICD-10). As the database contains de-identified and aggregated information, ethical approval and informed consent were not required. The database is available at https://wonder.cdc.gov.

This retrospective study assessed all-cause mortality trends from 1999 to 2024 among adults aged ≥25 years with both urinary tract cancers (ICD-10: C64–C68) and diabetes mellitus (ICD-10: E10–E14) recorded on death certificates. Records with missing key demographic information (age, sex, or race) were excluded.

### 2.2 Data extraction and subgroup analyses

We extracted information on annual death counts, corresponding population estimates, and demographic variables. Stratified analyses were conducted by age (25–34, 35–44, 45–54, 55–64, 65–74, 75–84, and ≥85 years), sex, and race (Hispanic, non-Hispanic White, non-Hispanic Black, and non-Hispanic Other). Geographic variation was assessed across four U.S. Census regions: Northeast, Midwest, South, and West. Urbanization status was classified according to the National Center for Health Statistics Urban–Rural Classification Scheme. Metropolitan areas included large central, large fringe, medium, and small metropolitan counties, whereas nonmetropolitan areas comprised micropolitan and noncore counties. This stratified framework enabled a detailed evaluation of mortality patterns across demographic, regional, and urban–rural contexts.

### 2.3 Statistical analysis

Crude mortality rates were calculated by dividing the annual number of deaths attributable to coexisting diabetes mellitus and urinary tract cancers by the corresponding U.S. population for each year. Age-adjusted mortality rates (AAMRs) were estimated using the direct standardization method, with the 2000 U.S. standard population as the reference (11). Temporal trends were assessed using the Joinpoint Regression Program (version 5.4.0.0; National Cancer Institute). Annual percent change (APC) and average annual percent change (AAPC), along with their 95% confidence intervals, were estimated using Monte Carlo permutation methods within the Joinpoint framework (see **Supplementary Tables 1**–**4**). Trends were considered statistically significant when the slope differed from zero based on two-sided t-tests (*P* < 0.05). This analytical strategy enabled a comprehensive evaluation of mortality trends across multiple demographic, geographic, and urbanization subgroups among individuals with coexisting urinary tract malignancies and diabetes mellitus.

## 3 Results

### 3.1 Overall characteristics

**Figure 1:**
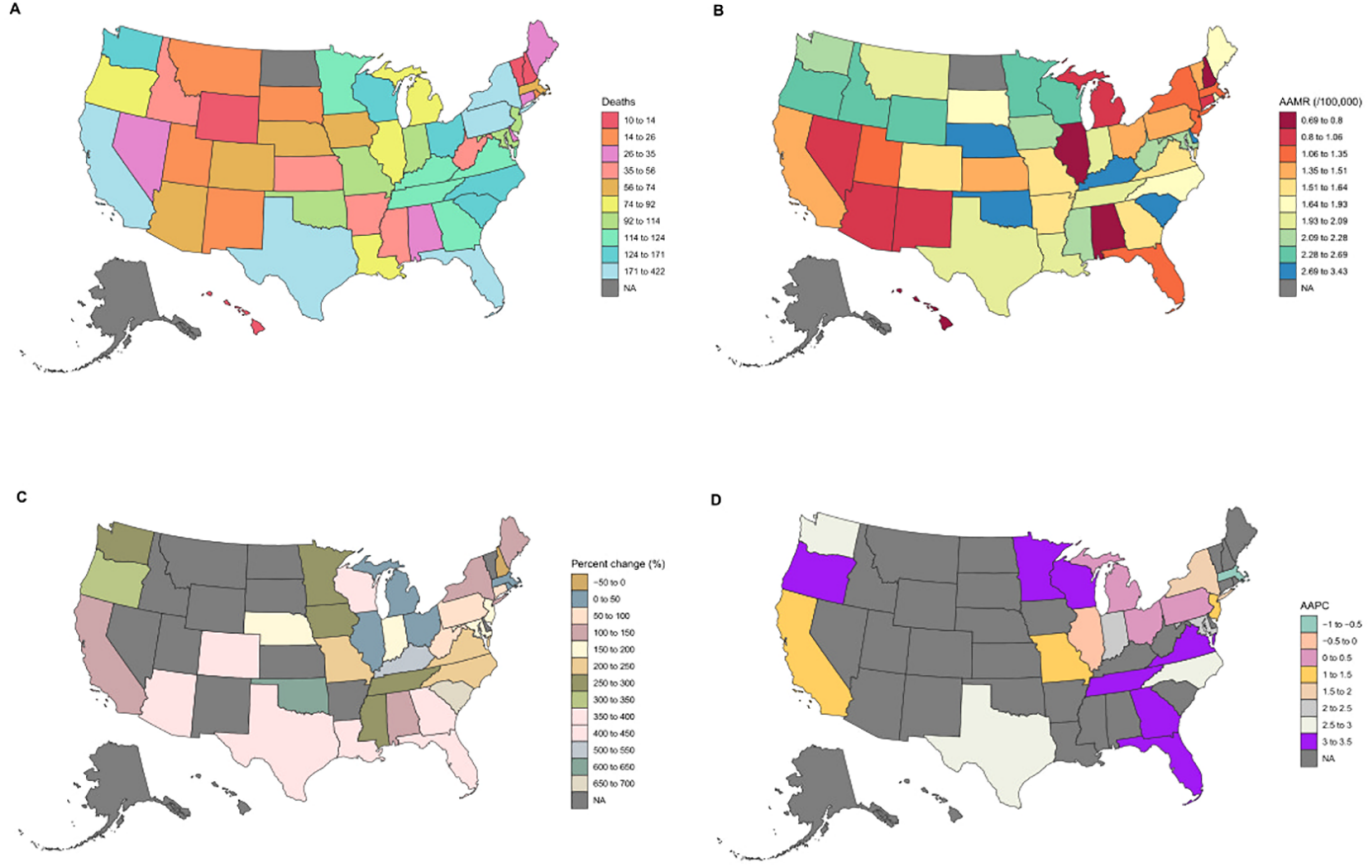
State-specific trends in urinary tract cancer and diabetes related mortality in the U.S. from 1999–2023: **(A)** deaths; **(B)** AAMRs; **(C)** percentage change; **(D)** AAPC.

During 1999–2024, mortality due to urinary tract cancer and diabetes mellitus in the United States increased substantially, with the annual number of deaths rising from 1, 566 in 1999 to 4, 487 in 2024 (**Figures 1A–D**; **Table 1**). The AAMR increased from 0.87 per 100, 000 population (95% CI: 0.83–0.91) to 1.53 per 100, 000 (95% CI: 1.49–1.58), corresponding to an overall upward trend with an AAPC of 2.30% (95% CI: 1.32%–3.29%). Joinpoint regression identified five distinct temporal segments. Mortality rose sharply from 1999 to 2001 (APC, 8.93%; 95% CI: 0.28%–18.33%), followed by a moderate but sustained increase from 2001 to 2011 (APC, 1.42%; 95% CI: 0.76%–2.09%). The trend remained relatively stable during 2011–2018 (APC, −0.06%; 95% CI: −1.13%–1.01%). A marked and statistically significant increase occurred between 2018 and 2021 (APC, 9.93%; 95% CI: 3.91%–16.29%), after which the trend showed a slight, non-significant decline during 2021–2024 (APC, −0.75%; 95% CI: −3.24%–1.80%).

**Table 1:** Trends in mortality and age-adjusted mortality rates for urinary tract cancer and diabetes mellitus in 1999 and 2024.

Characteristics	Deaths			AAMR		
	1999	2024	Percent change	1999	2024	AAPC (95% CI)
Overall	1566	4487	186.53	0.87 (0.83 to 0.91)	1.53 (1.49 to 1.58)	2.30 (1.32 to 3.29)*
Sex						
Female	522	1151	120.50	0.51 (0.46 to 0.55)	0.71 (0.67 to 0.76)	1.00 (-0.61 to 2.65)
Male	1044	3336	219.54	1.48 (1.39 to 1.57)	2.63 (2.53 to 2.72)	2.42 (1.66 to 3.17)*
Census Region						
Northeast	350	649	85.43	0.93 (0.83 to 1.03)	1.22 (1.12 to 1.32)	1.00 (-0.56 to 2.58)
Midwest	434	942	117.05	1.02 (0.93 to 1.12)	1.53 (1.44 to 1.64)	1.51 (-0.19 to 3.25)
South	458	1944	324.45	0.72 (0.66 to 0.79)	1.75 (1.67 to 1.83)	3.57 (2.41 to 4.74)*
West	324	952	193.83	0.92 (0.82 to 1.02)	1.45 (1.36 to 1.55)	1.68 (1.33 to 2.03)*
Races						
Hispanic	76	375	393.42	0.86 (0.67 to 1.09)	1.24 (1.11 to 1.38)	1.01 (0.49 to 1.53)*
NH Black	150	456	204.00	1.04 (0.87 to 1.21)	1.69 (1.53 to 1.85)	1.51 (0.55 to 2.48)*
NH White	1310	3488	166.26	0.87 (0.82 to 0.91)	1.63 (1.57 to 1.68)	2.51 (1.55 to 3.47)*
NH Other	29	161	455.17	0.69 (0.45 to 1.00)	0.78 (0.66 to 0.91)	0.81 (-0.04 to 1.66)
Urbanization						
Metropolitan	1230	3068	149.43	0.88 (0.83 to 0.92)	1.34 (1.29 to 1.39)	1.85 (1.22 to 2.48)*
Nonmetropolitan	336	855	154.46	0.99 (0.88 to 1.10)	1.92 (1.79 to 2.06)	3.19 (1.95 to 4.45)*
Age groups						
25–34 years			NA	NA (NA to NA)	NA (NA to NA)	
35–44 years		12	NA	NA (NA to NA)	0.03 (0.01 to 0.05)	
45–54 years	53	95	79.25	0.14 (0.11 to 0.19)	0.23 (0.19 to 0.28)	2.28 (1.17 to 3.41)*
55–64 years	162	425	162.35	0.68 (0.58 to 0.79)	1.02 (0.92 to 1.12)	1.28 (0.57 to 1.99)*
65–74 years	430	1131	163.02	2.33 (2.11 to 2.56)	3.19 (3.00 to 3.38)	1.27 (-0.05 to 2.60)
75–84 years	640	1734	170.94	5.24 (4.83 to 5.64)	8.98 (8.56 to 9.41)	2.15 (1.05 to 3.27)*
85+ years	274	1089	297.45	6.60 (5.82 to 7.38)	16.92 (15.92 to 17.93)	3.61 (1.65 to 5.60)*

* indicates statistically significant AAPC.

Urbanization-specific AAMR for 2023 is based on 2020 data. AAPC was calculated for 1999–2023.

Age-group AAMR and APC are calculated using crude rates.Age adjusted mortality rate (AAMR), confidence interval (CI), average annual percentage change (AAPC), non-Hispanic (NH).

### 3.2 Sex-stratified analyses

**Figure 2:**
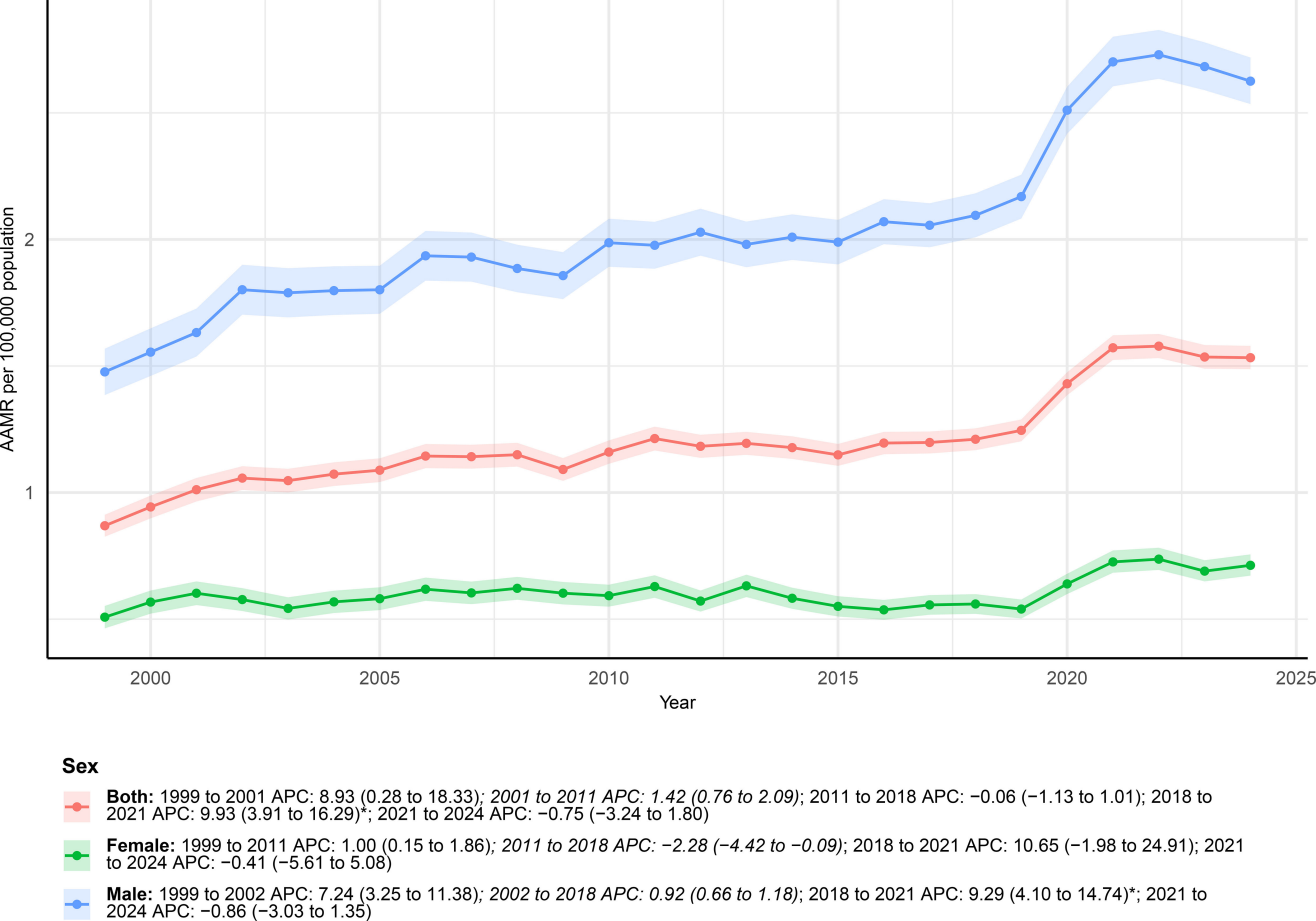
AAMRs of deaths with both urinary tract cancer and diabetes as underlying causes stratified by sex from 1999–2024.

Stratified by sex, mortality trends diverged considerably between males and females (**Figure 2**). Among females, the number of deaths rose from 522 in 1999 to 1, 151 in 2024, and the AAMR increased from 0.51 (95% CI: 0.46–0.55) to 0.71 (95% CI: 0.67–0.76) per 100, 000. The overall trend was not statistically significant (AAPC, 1.00%; 95% CI: −0.61% to 2.65%). Joinpoint analysis revealed a modest rise from 1999 to 2011 (APC, 1.00%; 95% CI: 0.15%–1.86%), followed by a significant decline during 2011–2018 (APC, −2.28%; 95% CI: −4.42% to −0.09%). A sharp but non-significant increase was observed between 2018 and 2021 (APC, 10.65%; 95% CI: −1.98% to 24.91%), and the trend thereafter remained stable from 2021 to 2024 (APC, −0.41%; 95% CI: −5.61% to 5.08%).

In contrast, males experienced a more pronounced rise in mortality burden. Deaths increased from 1, 044 in 1999 to 3, 336 in 2024, and the AAMR rose from 1.48 (95% CI: 1.39–1.57) to 2.63 (95% CI: 2.53–2.72) per 100, 000, showing a significant overall upward trend (AAPC, 2.42%; 95% CI: 1.66%–3.17%). Joinpoint analysis indicated a rapid increase during 1999–2002 (APC, 7.24%; 95% CI: 3.25%–11.38%), followed by a long period of gradual growth from 2002 to 2018 (APC, 0.92%; 95% CI: 0.66%–1.18%). A further significant increase was seen between 2018 and 2021 (APC, 9.29%; 95% CI: 4.10%–14.74%), after which the trend slightly declined during 2021–2024, though this decrease was not statistically significant (APC, −0.86%; 95% CI: −3.03% to 1.35%).

### 3.3 Age-stratified analyses

**Figure 3:**
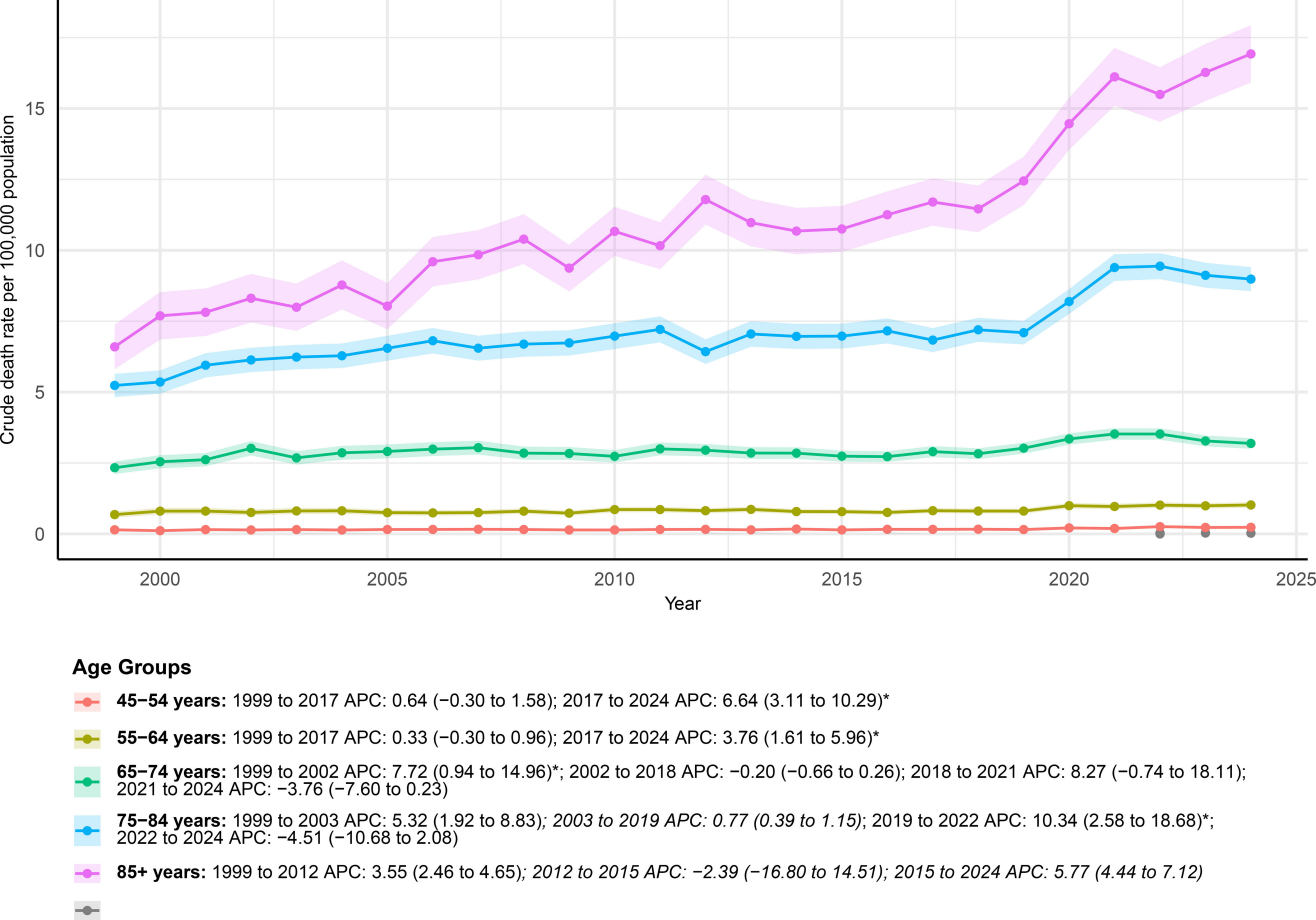
AAMRs of deaths with both urinary tract cancer and diabetes as underlying causes stratified by age group from 1999–2024.

Age-stratified analyses showed that mortality rose markedly with older age (**Figure 3**; **Supplementary Table 2**). The highest AAMRs were consistently seen in individuals aged 75 years and older, especially those 85 years or older, where the rate increased from 6.60 (95% CI: 5.82–7.38) per 100, 000 in 1999 to 16.92 (95% CI: 15.92–17.93) per 100, 000 in 2024. This group also had the largest overall increase (AAPC 3.61%; 95% CI: 1.65%–5.60%). A similar but less pronounced upward trend was observed among those aged 75–84 years (AAPC 2.15%; 95% CI: 1.05%–3.27%).

Among middle-aged adults, individuals aged 45–54 and 55–64 years also showed significant increases in AAMR (AAPC 2.28% and 1.28%, respectively). Joinpoint analysis further indicated that these increases were mainly driven by recent accelerations after 2017, with significant rises during 2017–2024 for both the 45–54 years group (APC 6.64%; 95% CI: 3.11%–10.29%) and the 55–64 years group (APC 3.76%; 95% CI: 1.61%–5.96%).

In older adults aged 65–74 and 75–84 years, temporary significant increases were observed in earlier or recent periods, including 1999–2002 for those aged 65–74 years (APC 7.72%; 95% CI: 0.94%–14.96%) and 2019–2022 for those aged 75–84 years (APC 10.34%; 95% CI: 2.58%–18.68%). By contrast, trends among younger age groups (<45 years) were unstable or could not be reliably estimated due to small case numbers.

### 3.4 Regional-stratified analyses

#### 3.4.1 Census regions stratified

**Figure 4:**
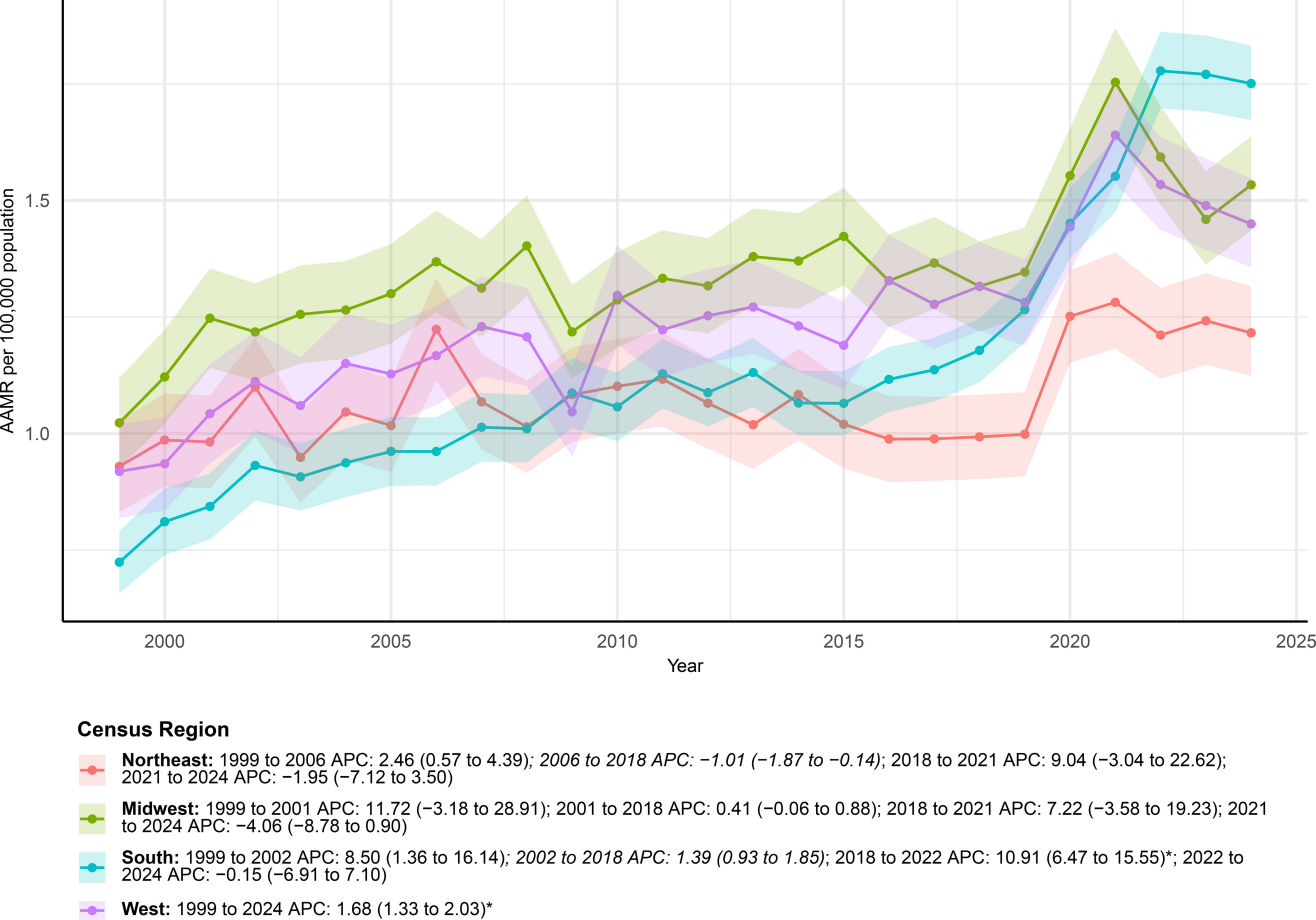
AAMRs of deaths with both urinary tract cancer and diabetes as underlying causes stratified by census region from 1999–2024.

Regional analyses revealed substantial geographic differences in mortality trends (**Figure 4**; **Table 1**). The largest increase was seen in the South, where deaths rose from 458 in 1999 to 1, 944 in 2024. The AAMR in this region increased from 0.72 (95% CI: 0.66–0.79) per 100, 000 to 1.75 (95% CI: 1.67–1.83) per 100, 000 with a significant overall upward trend (AAPC 3.57%; 95% CI: 2.41%–4.74%). Joinpoint analysis showed that this rise was largely driven by a sharp surge during 2018–2022 (APC 10.91%; 95% CI: 6.47%–15.55%). In the West, mortality also increased significantly over the study period. The AAMR rose from 0.92 (95% CI: 0.82–1.02) to 1.45 (95% CI: 1.36–1.55) per 100, 000 with a consistent upward trend (AAPC 1.68%; 95% CI: 1.33%–2.03%). Joinpoint regression further confirmed a steady increase throughout the entire period (APC 1.68%; 95% CI: 1.33%–2.03%). By contrast, the Northeast and Midwest showed more modest and non-significant changes over time. Although both regions had some fluctuations across subperiods, including early increases followed by later declines, their overall trends were not statistically significant (AAPC 1.00% and 1.51% respectively).

#### 3.4.2 State-stratified

State-level analyses revealed substantial heterogeneity in mortality trends across the United States (**Supplementary Table 1**). Overall, most states showed increasing AAMRs over the study period, though the magnitude and statistical significance of these trends varied widely. Significant upward trends were seen in several states, especially in the South and West. Notable increases occurred in Florida (AAPC 3.29%; 95% CI: 0.08%–6.61%), Georgia (AAPC 3.37%; 95% CI: 1.62%–5.15%), and Virginia (AAPC 3.05%; 95% CI: 1.62%–4.49%), as well as in western states such as Washington (AAPC 2.50%; 95% CI: 1.82%–3.19%) and Oregon (AAPC 3.41%; 95% CI: 2.32%–4.51%). In the Midwest, significant increases were also observed in Minnesota (AAPC 3.12%; 95% CI: 2.51%–3.74%) and Wisconsin (AAPC 3.31%; 95% CI: 2.36%–4.26%). By contrast, a few states showed stable or declining trends. For instance, Massachusetts had a slight but statistically significant decrease (AAPC −0.83%; 95% CI: −1.64% to −0.01%), while several states, including Illinois and Ohio, showed non-significant changes over time.

### 3.5 Race-stratified analyses

**Figure 5:**
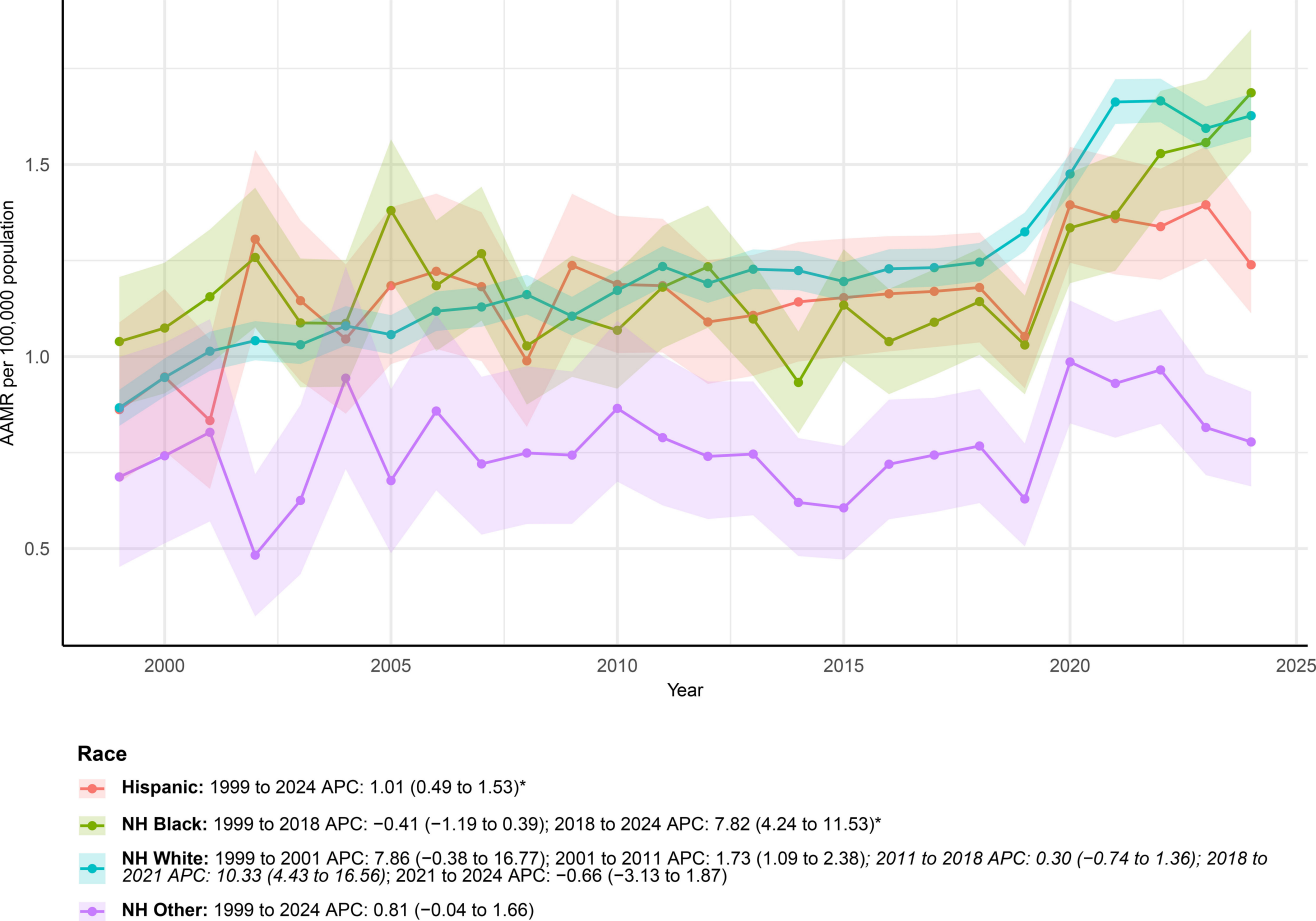
AAMRs of deaths with both urinary tract cancer and diabetes as underlying causes stratified by race from 1999–2024.

Racial disparities in mortality trends were evident (**Figure 5**; **Table 1**). Significant increases in AAMR were observed among Hispanic, non-Hispanic Black, and non-Hispanic White populations. For Hispanics, the AAMR rose from 0.86 (95% CI: 0.67–1.09) in 1999 to 1.24 (95% CI: 1.11–1.38) in 2024, with a steady upward trend over the entire period (AAPC 1.01%; 95% CI: 0.49%–1.53%).

Among non-Hispanic Black individuals, the overall increase was also significant (AAPC 1.51%; 95% CI: 0.55%–2.48%). Joinpoint analysis indicated that this rise was mainly driven by a marked acceleration during 2018–2024 (APC 7.82%; 95% CI: 4.24%–11.53%). For non-Hispanic White individuals, mortality increased significantly as well, with the AAMR rising from 0.87 (95% CI: 0.82–0.91) to 1.63 (95% CI: 1.57–1.68). This group had the highest overall growth among racial groups (AAPC 2.51%; 95% CI: 1.55%–3.47%). By contrast, the non-Hispanic Other group showed a non-significant change over the study period (AAPC 0.81%; 95% CI: −0.04% to 1.66%), despite showing the largest increase in the number of deaths among racial groups.

### 3.6 Urbanization-stratified analyses

**Figure 6:**
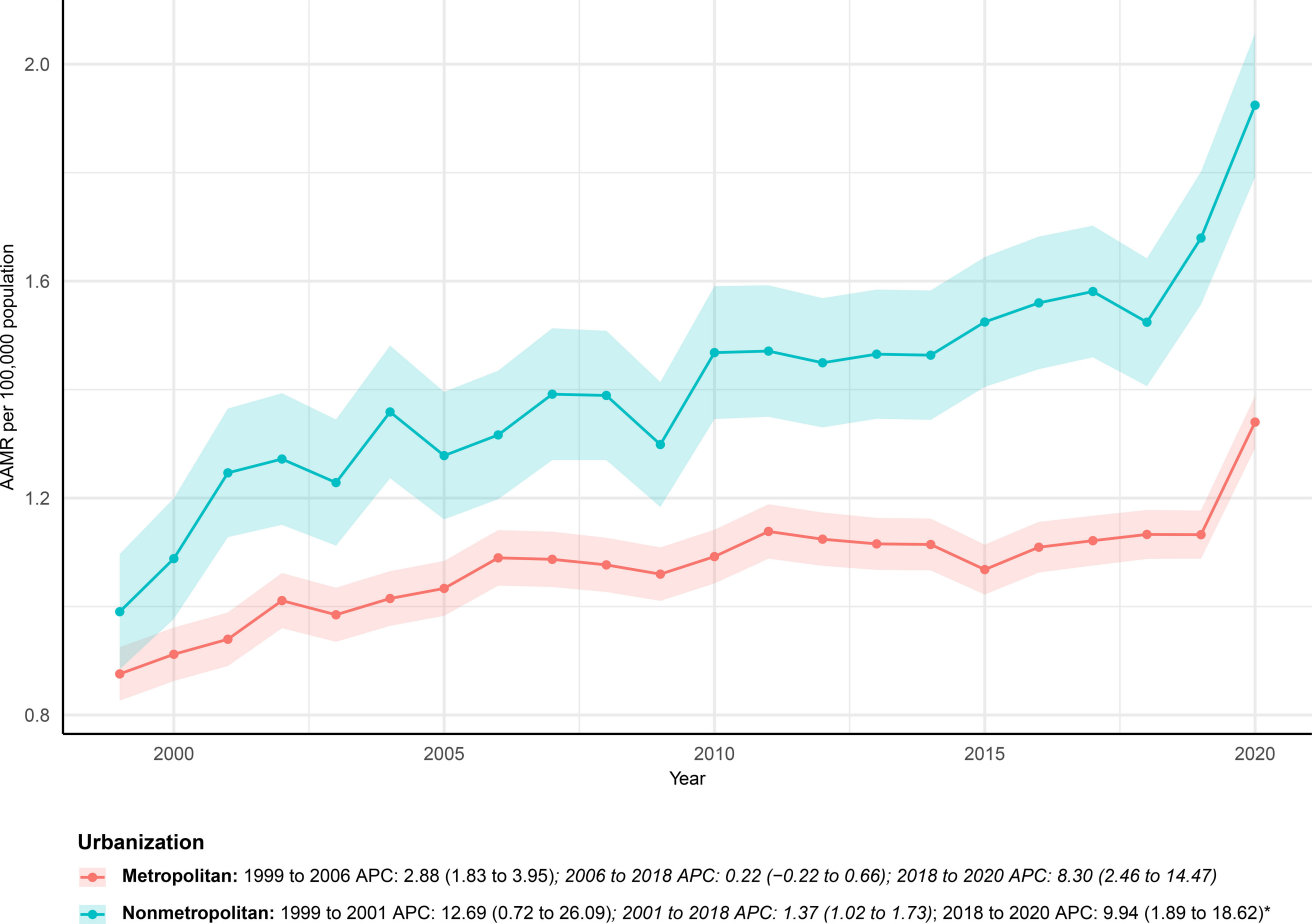
AAMRs of deaths with both urinary tract cancer and diabetes as underlying causes stratified by urbanization from 1999–2024. Data for urbanization AAMRs was unavailable for 2021–2024.

Urban–rural stratification showed that nonmetropolitan areas consistently had higher mortality burdens and faster increases than metropolitan areas (**Figure 6**). In metropolitan areas, deaths rose from 1, 230 in 1999 to 3, 068 in 2024, and the AAMR increased from 0.88 (95% CI: 0.83–0.92) to 1.34 (95% CI: 1.29–1.39) per 100, 000, reflecting a significant but moderate upward trend (AAPC 1.85%; 95% CI: 1.22%–2.48%). By comparison, nonmetropolitan areas experienced a steeper rise, with deaths climbing from 336 to 855 and the AAMR increasing from 0.99 (95% CI: 0.88–1.10) to 1.92 (95% CI: 1.79–2.06) per 100, 000, yielding a notably higher AAPC (3.19%; 95% CI: 1.95%–4.45%). Joinpoint analysis further revealed a significant acceleration in mortality during 2018–2020 in both metropolitan and nonmetropolitan areas (APC 9.94%; 95% CI: 1.89%-18.62% vs 8.3%; 95% CI: 2.46%-14.47%, respectively), reflecting a steeper rise observed in nonmetropolitan areas.

### 3.7 Subgroup analyses of mortality trends in different urinary cancers combined with diabetes

**Figure 7:**
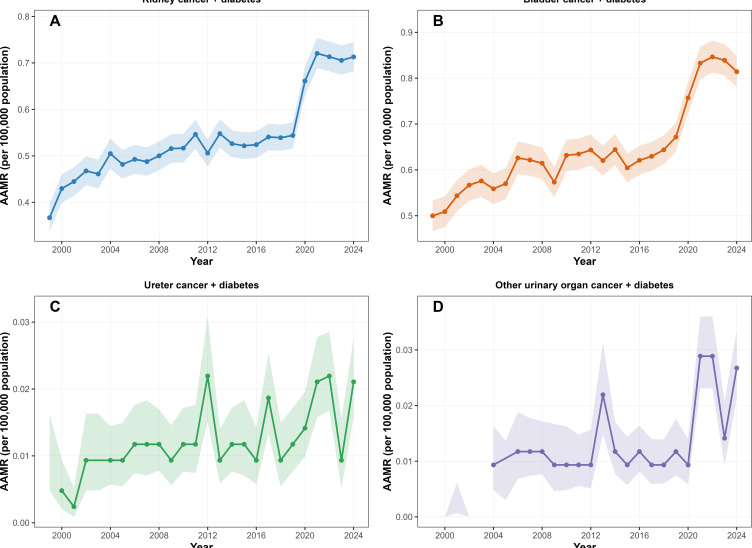
AAMRs of deaths of different urinary tract cancer and diabetes from 1999-2024. **(A)** Kidney cancer; **(B)** Bladder cancer; **(C)** Ureter cancer; **(D)** Other urinary cancer.

Subgroup analyses were also conducted for urinary tract cancer-specific mortality in patients with diabetes (**Figure 7**), encompassing malignant neoplasm of the kidney (C64), renal pelvis (C65), ureter (C66), bladder (C67), and other and unspecified urinary organs (C68). For C65 (malignant neoplasm of the renal pelvis), a considerable number of annual mortality estimates were either suppressed or flagged as unreliable in the CDC WONDER database, thereby precluding a meaningful assessment of temporal trends; consequently, this subtype was excluded from the Joinpoint regression analyses. For the remaining subtypes (C64, C66, C67, and C68), site-specific Joinpoint trends are shown in **Figures 7A–D**. The mortality patterns for kidney cancer (C64) and bladder cancer (C67) with diabetes were broadly consistent with the overall trend presented in **Figures 7A, B**, showing a relatively stable trajectories over majority of the study period. In contrast, ureter cancer (C66) and cancers of other and unspecified urinary organs (C68) demonstrated considerable annual fluctuations, characterized by instability across several calendar years—likely due to small case numbers and data suppression in the underlying database.

## 4 Discussion

In this nationwide analysis, mortality attributable to the co-occurrence of diabetes mellitus and urinary tract cancer showed a steady increase from 1999 to 2024, with notable disparities across demographic and geographic subgroups. The overall upward trend likely reflects the growing prevalence of diabetes alongside persistently high incidence rates of urinary tract malignancies, compounded by their synergistic effects on disease progression and patient survival. Diabetes is known to promote carcinogenesis and worsen cancer prognosis through mechanisms such as hyperinsulinemia, chronic inflammation, and oxidative stress—factors that may collectively contribute to the observed increase in mortality (12).

A marked sex disparity was evident, with males exhibiting both higher mortality rates and a steeper upward trajectory compared with females. This pattern aligns with established epidemiological data indicating that urinary tract cancers, particularly those of the bladder and kidney, are substantially more common in men, with incidence rates three to four times higher than in women (13). Additionally, men tend to have a higher burden of metabolic risk factors and are more frequently exposed to carcinogens, including tobacco smoke and occupational hazards (14). These combined factors likely amplify the adverse effects of diabetes on tumor progression and survival, contributing to the greater mortality burden observed among males.

Mortality increased substantially with age, with the highest rates and the most rapid increases occurring among individuals aged 85 years and older. This finding is biologically plausible, as aging is associated with cumulative carcinogen exposure, progressive declines in renal and metabolic function, and a higher prevalence of multimorbidity (15). Older patients are also less likely to receive aggressive oncologic treatment owing to frailty and diminished physiological reserve, which may further compromise survival (16). The presence of diabetes may exacerbate these vulnerabilities by impairing immune function and increasing susceptibility to treatment-related complications, thereby contributing to disproportionately high mortality in the oldest age groups.

Geographic variation was substantial, with the South experiencing the most pronounced increase in mortality in recent years. This regional pattern likely reflects the higher prevalence of diabetes, obesity, and socioeconomic disadvantage in the Southern United States, along with disparities in healthcare access and preventive services (17, 18). In contrast, the more modest trends observed in the Midwest and Northeast may indicate better control of risk factors and more robust healthcare infrastructure (19). These findings suggest that regional differences in population health and healthcare delivery play a critical role in shaping mortality outcomes.

Racial disparities were also evident, with non-Hispanic White individuals showing the largest overall increase in mortality. This may be partially explained by the higher baseline incidence of urinary tract cancers, particularly bladder cancer, in this population (20). Differences in lifestyle-related exposures such as smoking, as well as variations in comorbidity profiles and competing risks, may further contribute to this pattern (21). Although non-Hispanic Black and Hispanic populations also experienced rising mortality rates, the magnitude of change was comparatively smaller, suggesting heterogeneity in the interaction between diabetes and cancer across racial groups.

Urban–rural differences further highlighted the role of structural determinants of health. Nonmetropolitan areas consistently had higher mortality rates and steeper increases than metropolitan areas, likely reflecting limited access to specialized care, greater travel distances to healthcare facilities, and lower rates of early detection and disease management (22). Rural populations also tend to have a higher prevalence of behavioral risk factors and lower health literacy, which may delay diagnosis and worsen outcomes in patients with concurrent diabetes and cancer.

A notable sharp increase in mortality occurred after 2018, followed by a modest decline after 2021. This temporal pattern may be linked to disruptions in healthcare delivery during the COVID-19 pandemic. Widespread delays in cancer screening, diagnosis, and treatment, as well as interruptions in chronic disease management, have been documented during this period and may have disproportionately affected patients with diabetes and malignancies (23). Moreover, individuals with these comorbidities are known to be at increased risk of severe outcomes following SARS-CoV-2 infection, which may have contributed to excess mortality (24). The subsequent decline after 2021 may reflect the gradual restoration of healthcare services and adaptations in clinical care delivery.

Our subgroup analyses reveal heterogeneity that is often overlooked when urinary tract cancers are studied as a single group. The overall mortality pattern in patients with both urinary tract cancer and diabetes was largely driven by kidney and bladder cancers, the two most common subtypes with stable temporal trends. This suggests that the diabetes-associated mortality burden is not evenly distributed across all urinary tract tumors but instead concentrated in specific, high-incidence types. Clinically, this implies that the interaction between diabetes and cancer outcomes may be more relevant for common tumors rather than reflecting a generalized effect. Less common subtypes, such as ureter cancer and other rare urinary tract malignancies, showed pronounced temporal instability, likely due to small case numbers and statistical variability. This limits the reliability of population-level inferences for these rare subtypes. Importantly, treating heterogeneous tumor entities as a single group can obscure meaningful site-specific patterns and lead to oversimplified conclusions. Thus, our findings support a tumor-specific approach when examining cancer–diabetes interactions, especially in large-scale studies where rare cancers may contribute more noise than signal.

Several limitations should be acknowledged. As a retrospective analysis of death certificate data from CDC WONDER, this study is subject to potential misclassification bias stemming from ICD-10 coding practices and underreporting of diabetes and urinary tract cancers as contributing causes. The absence of detailed clinical data—including laboratory parameters, cancer staging, and treatment details—precludes assessment of disease severity and the impact of therapeutic interventions. Furthermore, the lack of information on socioeconomic status and healthcare access limits our ability to fully account for the observed disparities across demographic and geographic subgroups. Owing to data constraints, we were unable to examine whether racial disparities in mortality among patients with coexisting diabetes and urinary tract cancers persist uniformly across states. As an ecological study, these findings do not establish causality, highlighting the need for prospective studies that incorporate comprehensive clinical, social, and geographically stratified data.

## 5 Conclusion

From 1999 to 2024, mortality related to coexisting urinary tract cancers and diabetes mellitus rose in the United States, with a marked acceleration in recent years. Considerable disparities existed across subgroups, with higher burdens seen in males, older adults, residents of nonmetropolitan areas, and populations living in the South and West. These results point to a rising and unevenly distributed mortality burden, emphasizing the need for focused prevention efforts and integrated management strategies among high-risk groups.

## Data Availability

Publicly available datasets were analyzed in this study. This data can be found here: https://wonder.cdc.gov/.
