# Supplementary material for: Mortality trends in the co-occurrence of urinary tract cancer and diabetes mellitus
Source: Front Endocrinol (Lausanne). 2026 May 22;17:1843044. doi: 10.3389/fendo.2026.1843044 (PMC13236562; doi:10.3389/fendo.2026.1843044)
Supplement: Supplementary file 1 [file Table1.docx]

Supplementary table 1. State-specific Mortality and Age-Adjusted Mortality Rates for Concurrent Urinary Tract Cancer and Diabetes Mellitus in 1999 and 2024.

| States | Deaths | | | AAMR | | |
| --- | --- | --- | --- | --- | --- | --- |
|  | 1999 | 2024 | Percent Change | 1999 | 2024 | AAPC (95% CI) |
| Alabama | 15 | 34 | 126.67 | NA (0.29 to 0.86) | 0.77 (0.53 to 1.10) |  |
| Alaska |  |  | NA | NA (NA to NA) | NA (NA to NA) |  |
| Arizona | 13 | 70 | 438.46 | NA (0.22 to 0.69) | 0.99 (0.77 to 1.26) |  |
| Arkansas |  | 40 | NA | NA (NA to NA) | 1.52 (1.09 to 2.11) |  |
| California | 195 | 422 | 116.41 | 1.07 (0.92 to 1.22) | 1.35 (1.22 to 1.48) | 1.00 (0.09 to 1.93)* |
| Colorado | 16 | 73 | 356.25 | NA (0.44 to 1.25) | 1.63 (1.27 to 2.06) |  |
| Connecticut | 17 | 28 | 64.71 | NA (0.41 to 1.12) | 0.80 (0.53 to 1.19) |  |
| Delaware |  | 30 | NA | NA (NA to NA) | 2.96 (1.97 to 4.41) |  |
| District of Columbia |  |  | NA | NA (NA to NA) | NA (NA to NA) |  |
| Florida | 59 | 268 | 354.24 | 0.42 (0.32 to 0.55) | 1.07 (0.95 to 1.22) | 3.29 (0.08 to 6.61)* |
| Georgia | 25 | 122 | 388 | 0.63 (0.41 to 0.93) | 1.51 (1.24 to 1.81) | 3.37 (1.62 to 5.15)* |
| Hawaii |  | 11 | NA | NA (NA to NA) | 0.69 (0.34 to 1.37) |  |
| Idaho |  | 42 | NA | NA (NA to NA) | 2.67 (1.92 to 3.67) |  |
| Illinois | 72 | 82 | 13.89 | 0.93 (0.73 to 1.17) | 0.76 (0.60 to 0.95) | -0.20 (-0.75 to 0.34) |
| Indiana | 39 | 112 | 187.18 | 1.02 (0.73 to 1.40) | 1.93 (1.58 to 2.34) | 2.05 (1.40 to 2.71)* |
| Iowa | 18 | 65 | 261.11 | NA (0.49 to 1.29) | 2.25 (1.73 to 2.91) |  |
| Kansas |  | 35 | NA | NA (NA to NA) | 1.40 (0.97 to 1.99) |  |
| Kentucky | 19 | 115 | 505.26 | NA (0.44 to 1.15) | 2.90 (2.39 to 3.52) |  |
| Louisiana | 16 | 76 | 375 | NA (0.35 to 0.99) | 2.02 (1.58 to 2.55) |  |
| Maine | 13 | 29 | 123.08 | NA (0.75 to 2.41) | 1.88 (1.25 to 2.82) |  |
| Maryland | 39 | 113 | 189.74 | 1.27 (0.90 to 1.74) | 2.09 (1.72 to 2.54) | 2.32 (0.63 to 4.04)* |
| Massachusetts | 46 | 66 | 43.48 | 1.03 (0.76 to 1.38) | 1.06 (0.82 to 1.37) | -0.83 (-1.64 to -0.01)* |
| Michigan | 75 | 91 | 21.33 | 1.24 (0.97 to 1.55) | 1.00 (0.81 to 1.24) | 0.10 (-0.69 to 0.89) |
| Minnesota | 31 | 114 | 267.74 | 1.00 (0.68 to 1.41) | 2.29 (1.89 to 2.76) | 3.12 (2.51 to 3.74)* |
| Mississippi | 15 | 54 | 260 | NA (0.49 to 1.43) | 2.16 (1.62 to 2.86) |  |
| Missouri | 29 | 92 | 217.24 | 0.75 (0.50 to 1.07) | 1.63 (1.31 to 2.01) | 1.08 (0.23 to 1.93)* |
| Montana |  | 22 | NA | NA (NA to NA) | 1.99 (1.24 to 3.15) |  |
| Nebraska | 20 | 56 | 180 | 1.64 (1.00 to 2.54) | 3.43 (2.59 to 4.50) |  |
| Nevada |  | 26 | NA | NA (NA to NA) | 1.00 (0.65 to 1.50) |  |
| New Hampshire | 14 | 10 | -28.57 | NA (1.02 to 3.13) | 0.71 (0.34 to 1.44) |  |
| New Jersey | 38 | 101 | 165.79 | 0.67 (0.48 to 0.93) | 1.22 (0.99 to 1.49) | 1.07 (0.13 to 2.02)* |
| New Mexico |  | 20 | NA | NA (NA to NA) | 1.02 (0.61 to 1.66) |  |
| New York | 91 | 217 | 138.46 | 0.75 (0.60 to 0.92) | 1.19 (1.04 to 1.37) | 2.00 (1.26 to 2.74)* |
| North Carolina | 47 | 159 | 238.3 | 0.98 (0.72 to 1.30) | 1.73 (1.47 to 2.03) | 2.80 (0.04 to 5.63)* |
| North Dakota | 12 |  | NA | NA (1.29 to 4.34) | NA (NA to NA) |  |
| Ohio | 105 | 149 | 41.9 | 1.38 (1.12 to 1.65) | 1.39 (1.17 to 1.65) | 0.22 (-1.33 to 1.80) |
| Oklahoma | 15 | 110 | 633.33 | NA (0.36 to 1.07) | 3.33 (2.73 to 4.04) |  |
| Oregon | 21 | 91 | 333.33 | 0.92 (0.57 to 1.41) | 2.33 (1.87 to 2.89) | 3.41 (2.32 to 4.51)* |
| Pennsylvania | 114 | 171 | 50 | 1.20 (0.98 to 1.42) | 1.35 (1.15 to 1.58) | 0.39 (-1.60 to 2.42) |
| Rhode Island | 13 | 17 | 30.77 | NA (0.88 to 2.84) | 1.65 (0.96 to 2.76) |  |
| South Carolina | 17 | 133 | 682.35 | NA (0.40 to 1.10) | 2.69 (2.24 to 3.21) |  |
| South Dakota |  | 14 | NA | NA (NA to NA) | 1.70 (0.92 to 3.04) |  |
| Tennessee | 33 | 117 | 254.55 | 0.93 (0.64 to 1.30) | 1.96 (1.62 to 2.37) | 3.17 (-0.13 to 6.57) |
| Texas | 88 | 411 | 367.05 | 0.85 (0.68 to 1.04) | 2.00 (1.81 to 2.20) | 2.53 (-0.22 to 5.35) |
| Utah |  | 23 | NA | NA (NA to NA) | 1.14 (0.72 to 1.75) |  |
| Vermont |  | 10 | NA | NA (NA to NA) | 1.46 (0.69 to 3.01) |  |
| Virginia | 35 | 116 | 231.43 | 0.88 (0.61 to 1.22) | 1.60 (1.32 to 1.94) | 3.05 (1.62 to 4.49)* |
| Washington | 34 | 134 | 294.12 | 0.98 (0.68 to 1.37) | 2.12 (1.77 to 2.52) | 2.50 (1.82 to 3.19)* |
| West Virginia | 21 | 39 | 85.71 | 1.50 (0.93 to 2.30) | 2.13 (1.51 to 3.01) |  |
| Wisconsin | 23 | 124 | 439.13 | 0.64 (0.41 to 0.97) | 2.28 (1.90 to 2.75) | 3.31 (2.36 to 4.26)* |
| Wyoming |  | 12 | NA | NA (NA to NA) | 2.43 (1.24 to 4.49) |  |

* indicates statistically significant AAPC.

#Age-group AAMR and APC are calculated using crude rates.

Abbreviations: Age adjusted mortality rate (AAMR), confidence interval (CI), average annual percentage change (AAPC), non-Hispanic (NH).

Supplementary table 2. Age-Adjusted Mortality Rates and Standard Errors by Characteristics Across Years.

| Characteristics | Year | AAMR | SE |
| --- | --- | --- | --- |
| Census Region_Midwest | 1999 | 1.022963022 | 0.049249761 |
| Census Region_Midwest | 2000 | 1.121503496 | 0.051315141 |
| Census Region_Midwest | 2001 | 1.247386537 | 0.054368645 |
| Census Region_Midwest | 2002 | 1.218002647 | 0.052940606 |
| Census Region_Midwest | 2003 | 1.255693853 | 0.053442535 |
| Census Region_Midwest | 2004 | 1.26490777 | 0.053414516 |
| Census Region_Midwest | 2005 | 1.300083107 | 0.054291754 |
| Census Region_Midwest | 2006 | 1.36861496 | 0.055877028 |
| Census Region_Midwest | 2007 | 1.311876232 | 0.053423177 |
| Census Region_Midwest | 2008 | 1.40244495 | 0.055194944 |
| Census Region_Midwest | 2009 | 1.218218868 | 0.05111956 |
| Census Region_Midwest | 2010 | 1.286649762 | 0.052129922 |
| Census Region_Midwest | 2011 | 1.332942499 | 0.052705698 |
| Census Region_Midwest | 2012 | 1.316830353 | 0.052132948 |
| Census Region_Midwest | 2013 | 1.379672413 | 0.052616428 |
| Census Region_Midwest | 2014 | 1.370171973 | 0.052122448 |
| Census Region_Midwest | 2015 | 1.422990111 | 0.053067161 |
| Census Region_Midwest | 2016 | 1.327548214 | 0.050658363 |
| Census Region_Midwest | 2017 | 1.365851216 | 0.050366276 |
| Census Region_Midwest | 2018 | 1.315812087 | 0.049195863 |
| Census Region_Midwest | 2019 | 1.346303935 | 0.048623596 |
| Census Region_Midwest | 2020 | 1.552986622 | 0.052041361 |
| Census Region_Midwest | 2021 | 1.753518456 | 0.056660191 |
| Census Region_Midwest | 2022 | 1.593041813 | 0.052898361 |
| Census Region_Midwest | 2023 | 1.459246648 | 0.050396578 |
| Census Region_Midwest | 2024 | 1.533408026 | 0.050622507 |
| Census Region_Northeast | 1999 | 0.929548201 | 0.049693507 |
| Census Region_Northeast | 2000 | 0.986090726 | 0.051016463 |
| Census Region_Northeast | 2001 | 0.981952904 | 0.050774601 |
| Census Region_Northeast | 2002 | 1.099670612 | 0.053589601 |
| Census Region_Northeast | 2003 | 0.948865012 | 0.049278408 |
| Census Region_Northeast | 2004 | 1.046216328 | 0.051757354 |
| Census Region_Northeast | 2005 | 1.017194578 | 0.050885398 |
| Census Region_Northeast | 2006 | 1.223673117 | 0.055664431 |
| Census Region_Northeast | 2007 | 1.068055351 | 0.052139243 |
| Census Region_Northeast | 2008 | 1.014503445 | 0.050174291 |
| Census Region_Northeast | 2009 | 1.08331889 | 0.051687922 |
| Census Region_Northeast | 2010 | 1.101278853 | 0.05145964 |
| Census Region_Northeast | 2011 | 1.116619037 | 0.051783377 |
| Census Region_Northeast | 2012 | 1.065106633 | 0.049442006 |
| Census Region_Northeast | 2013 | 1.018956847 | 0.048304327 |
| Census Region_Northeast | 2014 | 1.083697045 | 0.049975043 |
| Census Region_Northeast | 2015 | 1.020049381 | 0.048350186 |
| Census Region_Northeast | 2016 | 0.988179502 | 0.046988207 |
| Census Region_Northeast | 2017 | 0.988661621 | 0.045988224 |
| Census Region_Northeast | 2018 | 0.992839428 | 0.046082799 |
| Census Region_Northeast | 2019 | 0.998580553 | 0.045880509 |
| Census Region_Northeast | 2020 | 1.251496399 | 0.050463479 |
| Census Region_Northeast | 2021 | 1.281513062 | 0.051406993 |
| Census Region_Northeast | 2022 | 1.211179628 | 0.048815952 |
| Census Region_Northeast | 2023 | 1.24186805 | 0.048957709 |
| Census Region_Northeast | 2024 | 1.216012182 | 0.047950332 |
| Census Region_South | 1999 | 0.724126916 | 0.034030523 |
| Census Region_South | 2000 | 0.810920334 | 0.03613987 |
| Census Region_South | 2001 | 0.843699374 | 0.036064661 |
| Census Region_South | 2002 | 0.931615614 | 0.038187827 |
| Census Region_South | 2003 | 0.907226469 | 0.036950828 |
| Census Region_South | 2004 | 0.937193807 | 0.037550771 |
| Census Region_South | 2005 | 0.961650286 | 0.037693783 |
| Census Region_South | 2006 | 0.961573642 | 0.037197847 |
| Census Region_South | 2007 | 1.013485585 | 0.03762591 |
| Census Region_South | 2008 | 1.010286651 | 0.036979626 |
| Census Region_South | 2009 | 1.087132791 | 0.038179381 |
| Census Region_South | 2010 | 1.05724304 | 0.037451868 |
| Census Region_South | 2011 | 1.128338153 | 0.037995389 |
| Census Region_South | 2012 | 1.087892475 | 0.036618493 |
| Census Region_South | 2013 | 1.130811003 | 0.037589778 |
| Census Region_South | 2014 | 1.065530376 | 0.035349018 |
| Census Region_South | 2015 | 1.064814026 | 0.035071096 |
| Census Region_South | 2016 | 1.116270172 | 0.035473783 |
| Census Region_South | 2017 | 1.136838688 | 0.034766318 |
| Census Region_South | 2018 | 1.178441212 | 0.034984272 |
| Census Region_South | 2019 | 1.265856326 | 0.035771635 |
| Census Region_South | 2020 | 1.451162868 | 0.038081065 |
| Census Region_South | 2021 | 1.551874107 | 0.039884651 |
| Census Region_South | 2022 | 1.77797254 | 0.041565709 |
| Census Region_South | 2023 | 1.770516816 | 0.041073662 |
| Census Region_South | 2024 | 1.750756282 | 0.040305308 |
| Census Region_West | 1999 | 0.918878637 | 0.051193003 |
| Census Region_West | 2000 | 0.935105418 | 0.051012884 |
| Census Region_West | 2001 | 1.042607925 | 0.053800285 |
| Census Region_West | 2002 | 1.11145266 | 0.054931156 |
| Census Region_West | 2003 | 1.060017492 | 0.052434463 |
| Census Region_West | 2004 | 1.150253413 | 0.054649364 |
| Census Region_West | 2005 | 1.127838024 | 0.053582324 |
| Census Region_West | 2006 | 1.167065636 | 0.053926626 |
| Census Region_West | 2007 | 1.229411479 | 0.054693346 |
| Census Region_West | 2008 | 1.207390284 | 0.053434021 |
| Census Region_West | 2009 | 1.046521213 | 0.048918965 |
| Census Region_West | 2010 | 1.296489339 | 0.054424876 |
| Census Region_West | 2011 | 1.222322998 | 0.051461378 |
| Census Region_West | 2012 | 1.252769487 | 0.051061906 |
| Census Region_West | 2013 | 1.271313543 | 0.051244243 |
| Census Region_West | 2014 | 1.230772238 | 0.04970418 |
| Census Region_West | 2015 | 1.189428326 | 0.047794795 |
| Census Region_West | 2016 | 1.327924399 | 0.050199463 |
| Census Region_West | 2017 | 1.277267922 | 0.048532284 |
| Census Region_West | 2018 | 1.315459257 | 0.04849971 |
| Census Region_West | 2019 | 1.281380418 | 0.047056438 |
| Census Region_West | 2020 | 1.443407715 | 0.04880957 |
| Census Region_West | 2021 | 1.640222531 | 0.053626372 |
| Census Region_West | 2022 | 1.534145353 | 0.050110648 |
| Census Region_West | 2023 | 1.489028082 | 0.049106171 |
| Census Region_West | 2024 | 1.449575937 | 0.047795432 |
| Race_Hispanic | 1999 | 0.86187062 | 0.103046981 |
| Race_Hispanic | 2000 | 0.947077089 | 0.104627184 |
| Race_Hispanic | 2001 | 0.833209015 | 0.096316969 |
| Race_Hispanic | 2002 | 1.305482331 | 0.118306984 |
| Race_Hispanic | 2003 | 1.145452091 | 0.106666055 |
| Race_Hispanic | 2004 | 1.045476237 | 0.099117523 |
| Race_Hispanic | 2005 | 1.184484106 | 0.104145436 |
| Race_Hispanic | 2006 | 1.22189992 | 0.103144986 |
| Race_Hispanic | 2007 | 1.181918942 | 0.098896679 |
| Race_Hispanic | 2008 | 0.988839207 | 0.087743261 |
| Race_Hispanic | 2009 | 1.237129817 | 0.095159084 |
| Race_Hispanic | 2010 | 1.187754063 | 0.091144213 |
| Race_Hispanic | 2011 | 1.184550849 | 0.088704266 |
| Race_Hispanic | 2012 | 1.089958823 | 0.082255463 |
| Race_Hispanic | 2013 | 1.107182577 | 0.080302893 |
| Race_Hispanic | 2014 | 1.142283241 | 0.079201513 |
| Race_Hispanic | 2015 | 1.153418298 | 0.078183801 |
| Race_Hispanic | 2016 | 1.163414871 | 0.076477163 |
| Race_Hispanic | 2017 | 1.169665029 | 0.074072542 |
| Race_Hispanic | 2018 | 1.179707958 | 0.072966002 |
| Race_Hispanic | 2019 | 1.052432257 | 0.068917333 |
| Race_Hispanic | 2020 | 1.394848585 | 0.076868633 |
| Race_Hispanic | 2021 | 1.359199495 | 0.076180464 |
| Race_Hispanic | 2022 | 1.338248066 | 0.072471767 |
| Race_Hispanic | 2023 | 1.394995531 | 0.073372993 |
| Race_Hispanic | 2024 | 1.238925933 | 0.066146439 |
| Race_NH Black | 1999 | 1.039115153 | 0.085709897 |
| Race_NH Black | 2000 | 1.074180825 | 0.086741284 |
| Race_NH Black | 2001 | 1.155757192 | 0.089297377 |
| Race_NH Black | 2002 | 1.258030723 | 0.092626284 |
| Race_NH Black | 2003 | 1.087558299 | 0.085546569 |
| Race_NH Black | 2004 | 1.086243813 | 0.084037138 |
| Race_NH Black | 2005 | 1.380153468 | 0.09457116 |
| Race_NH Black | 2006 | 1.184520494 | 0.086836383 |
| Race_NH Black | 2007 | 1.267865472 | 0.088824121 |
| Race_NH Black | 2008 | 1.027698595 | 0.077942096 |
| Race_NH Black | 2009 | 1.105060495 | 0.080548601 |
| Race_NH Black | 2010 | 1.068309175 | 0.077497072 |
| Race_NH Black | 2011 | 1.180635184 | 0.081105107 |
| Race_NH Black | 2012 | 1.233915148 | 0.08106092 |
| Race_NH Black | 2013 | 1.097657433 | 0.075625275 |
| Race_NH Black | 2014 | 0.932497862 | 0.067558285 |
| Race_NH Black | 2015 | 1.133894963 | 0.074165137 |
| Race_NH Black | 2016 | 1.038917969 | 0.06982959 |
| Race_NH Black | 2017 | 1.089365474 | 0.070287285 |
| Race_NH Black | 2018 | 1.143106909 | 0.070580785 |
| Race_NH Black | 2019 | 1.030045161 | 0.065495957 |
| Race_NH Black | 2020 | 1.335041226 | 0.073506742 |
| Race_NH Black | 2021 | 1.36849896 | 0.076064486 |
| Race_NH Black | 2022 | 1.528458868 | 0.078694187 |
| Race_NH Black | 2023 | 1.557160433 | 0.079291823 |
| Race_NH Black | 2024 | 1.686845059 | 0.079907548 |
| Race_NH Other | 1999 | 0.686509719 | 0.132214875 |
| Race_NH Other | 2000 | 0.741680407 | 0.126832092 |
| Race_NH Other | 2001 | 0.802789993 | 0.128487679 |
| Race_NH Other | 2002 | 0.482964196 | 0.09032658 |
| Race_NH Other | 2003 | 0.625398514 | 0.106943777 |
| Race_NH Other | 2004 | 0.943654637 | 0.129042465 |
| Race_NH Other | 2005 | 0.676980193 | 0.104345821 |
| Race_NH Other | 2006 | 0.858134307 | 0.11260301 |
| Race_NH Other | 2007 | 0.720437903 | 0.101092748 |
| Race_NH Other | 2008 | 0.748875972 | 0.100755871 |
| Race_NH Other | 2009 | 0.74328371 | 0.097898406 |
| Race_NH Other | 2010 | 0.865044204 | 0.103457561 |
| Race_NH Other | 2011 | 0.788803488 | 0.095858749 |
| Race_NH Other | 2012 | 0.740100815 | 0.088773854 |
| Race_NH Other | 2013 | 0.745992852 | 0.086335918 |
| Race_NH Other | 2014 | 0.62016615 | 0.075873769 |
| Race_NH Other | 2015 | 0.606011788 | 0.072756297 |
| Race_NH Other | 2016 | 0.719692263 | 0.077277963 |
| Race_NH Other | 2017 | 0.74356327 | 0.075919388 |
| Race_NH Other | 2018 | 0.767122458 | 0.075808253 |
| Race_NH Other | 2019 | 0.629258388 | 0.066254247 |
| Race_NH Other | 2020 | 0.985964757 | 0.081588531 |
| Race_NH Other | 2021 | 0.930070047 | 0.075056851 |
| Race_NH Other | 2022 | 0.965166732 | 0.074282162 |
| Race_NH Other | 2023 | 0.81526404 | 0.065756619 |
| Race_NH Other | 2024 | 0.777673423 | 0.061314313 |
| Race_NH White | 1999 | 0.866636867 | 0.024014668 |
| Race_NH White | 2000 | 0.945598745 | 0.025186874 |
| Race_NH White | 2001 | 1.013731096 | 0.025988159 |
| Race_NH White | 2002 | 1.041298226 | 0.025996708 |
| Race_NH White | 2003 | 1.030828844 | 0.025690099 |
| Race_NH White | 2004 | 1.079771092 | 0.026247272 |
| Race_NH White | 2005 | 1.057043885 | 0.025910218 |
| Race_NH White | 2006 | 1.117998447 | 0.026198286 |
| Race_NH White | 2007 | 1.129089524 | 0.026336306 |
| Race_NH White | 2008 | 1.161267847 | 0.026344776 |
| Race_NH White | 2009 | 1.105127829 | 0.025719996 |
| Race_NH White | 2010 | 1.172119349 | 0.026333746 |
| Race_NH White | 2011 | 1.234439276 | 0.027055338 |
| Race_NH White | 2012 | 1.190547183 | 0.025934502 |
| Race_NH White | 2013 | 1.227359108 | 0.02626993 |
| Race_NH White | 2014 | 1.223818039 | 0.026039274 |
| Race_NH White | 2015 | 1.195365692 | 0.025774736 |
| Race_NH White | 2016 | 1.228423295 | 0.025901994 |
| Race_NH White | 2017 | 1.231533947 | 0.025324614 |
| Race_NH White | 2018 | 1.245829894 | 0.025469054 |
| Race_NH White | 2019 | 1.324624876 | 0.025627657 |
| Race_NH White | 2020 | 1.475375806 | 0.026942932 |
| Race_NH White | 2021 | 1.662786807 | 0.029368314 |
| Race_NH White | 2022 | 1.665594514 | 0.028670995 |
| Race_NH White | 2023 | 1.594202625 | 0.028215575 |
| Race_NH White | 2024 | 1.627141629 | 0.028023615 |
| Sex_Both | 1999 | 0.869035134 | 0.022048771 |
| Sex_Both | 2000 | 0.943200478 | 0.022924437 |
| Sex_Both | 2001 | 1.011332829 | 0.023675441 |
| Sex_Both | 2002 | 1.057188807 | 0.023920813 |
| Sex_Both | 2003 | 1.047143906 | 0.023783322 |
| Sex_Both | 2004 | 1.072836843 | 0.023790465 |
| Sex_Both | 2005 | 1.088370246 | 0.023910686 |
| Sex_Both | 2006 | 1.144264752 | 0.024279302 |
| Sex_Both | 2007 | 1.141750854 | 0.024001 |
| Sex_Both | 2008 | 1.149585361 | 0.023866418 |
| Sex_Both | 2009 | 1.091259332 | 0.022897766 |
| Sex_Both | 2010 | 1.160012382 | 0.023515835 |
| Sex_Both | 2011 | 1.213375977 | 0.023962729 |
| Sex_Both | 2012 | 1.18278195 | 0.023242513 |
| Sex_Both | 2013 | 1.194565026 | 0.023084664 |
| Sex_Both | 2014 | 1.177582909 | 0.022717465 |
| Sex_Both | 2015 | 1.148918336 | 0.022045444 |
| Sex_Both | 2016 | 1.195629213 | 0.022509505 |
| Sex_Both | 2017 | 1.197908881 | 0.022057736 |
| Sex_Both | 2018 | 1.210637544 | 0.021922728 |
| Sex_Both | 2019 | 1.245711295 | 0.02194951 |
| Sex_Both | 2020 | 1.42997166 | 0.023091265 |
| Sex_Both | 2021 | 1.571978514 | 0.024652192 |
| Sex_Both | 2022 | 1.578439954 | 0.02411859 |
| Sex_Both | 2023 | 1.535548708 | 0.023676253 |
| Sex_Both | 2024 | 1.532792267 | 0.023286173 |
| Sex_Female | 1999 | 0.507875106 | 0.022540608 |
| Sex_Female | 2000 | 0.567393261 | 0.023275622 |
| Sex_Female | 2001 | 0.602322089 | 0.023780219 |
| Sex_Female | 2002 | 0.577359546 | 0.023063241 |
| Sex_Female | 2003 | 0.54242775 | 0.022343249 |
| Sex_Female | 2004 | 0.568239286 | 0.022498338 |
| Sex_Female | 2005 | 0.580755694 | 0.022975768 |
| Sex_Female | 2006 | 0.618227711 | 0.02340639 |
| Sex_Female | 2007 | 0.603722476 | 0.022712173 |
| Sex_Female | 2008 | 0.62178679 | 0.022998591 |
| Sex_Female | 2009 | 0.602582643 | 0.022589285 |
| Sex_Female | 2010 | 0.59274703 | 0.022086356 |
| Sex_Female | 2011 | 0.628818661 | 0.022623338 |
| Sex_Female | 2012 | 0.571470478 | 0.021223336 |
| Sex_Female | 2013 | 0.631480449 | 0.022544148 |
| Sex_Female | 2014 | 0.582535233 | 0.021252256 |
| Sex_Female | 2015 | 0.55052281 | 0.020451934 |
| Sex_Female | 2016 | 0.536772912 | 0.019986811 |
| Sex_Female | 2017 | 0.556201593 | 0.019785396 |
| Sex_Female | 2018 | 0.559855326 | 0.019843714 |
| Sex_Female | 2019 | 0.539952838 | 0.019047688 |
| Sex_Female | 2020 | 0.639196357 | 0.020430144 |
| Sex_Female | 2021 | 0.726087396 | 0.022109677 |
| Sex_Female | 2022 | 0.737018511 | 0.02203508 |
| Sex_Female | 2023 | 0.689755213 | 0.021012577 |
| Sex_Female | 2024 | 0.712646349 | 0.021148469 |
| Sex_Male | 1999 | 1.476829483 | 0.046655941 |
| Sex_Male | 2000 | 1.554789516 | 0.047927074 |
| Sex_Male | 2001 | 1.632112429 | 0.048279509 |
| Sex_Male | 2002 | 1.801337549 | 0.050267451 |
| Sex_Male | 2003 | 1.789228204 | 0.049528245 |
| Sex_Male | 2004 | 1.797704794 | 0.049065919 |
| Sex_Male | 2005 | 1.801195595 | 0.04845608 |
| Sex_Male | 2006 | 1.935339595 | 0.050132975 |
| Sex_Male | 2007 | 1.930278542 | 0.049481363 |
| Sex_Male | 2008 | 1.885235539 | 0.047884695 |
| Sex_Male | 2009 | 1.857111079 | 0.047297117 |
| Sex_Male | 2010 | 1.986963612 | 0.048459913 |
| Sex_Male | 2011 | 1.976926081 | 0.047220233 |
| Sex_Male | 2012 | 2.02858949 | 0.047301192 |
| Sex_Male | 2013 | 1.98036656 | 0.045962713 |
| Sex_Male | 2014 | 2.009049143 | 0.045922251 |
| Sex_Male | 2015 | 1.989314579 | 0.044919022 |
| Sex_Male | 2016 | 2.070053029 | 0.045323713 |
| Sex_Male | 2017 | 2.056065932 | 0.044339622 |
| Sex_Male | 2018 | 2.095179501 | 0.044353282 |
| Sex_Male | 2019 | 2.16917498 | 0.044202667 |
| Sex_Male | 2020 | 2.510570442 | 0.047419128 |
| Sex_Male | 2021 | 2.701471875 | 0.049728572 |
| Sex_Male | 2022 | 2.729680652 | 0.048936953 |
| Sex_Male | 2023 | 2.682778497 | 0.047930228 |
| Sex_Male | 2024 | 2.625237095 | 0.046591765 |
| State_Alabama | 1999 | NA | 0.135284575 |
| State_Alabama | 2000 | 0.790256167 | 0.164956838 |
| State_Alabama | 2001 | NA | 0.138383025 |
| State_Alabama | 2002 | 0.807222592 | 0.164998395 |
| State_Alabama | 2003 | 0.731925791 | 0.156554351 |
| State_Alabama | 2004 | 0.844386175 | 0.16935992 |
| State_Alabama | 2005 | 0.962639031 | 0.176402523 |
| State_Alabama | 2006 | 0.953569808 | 0.17506327 |
| State_Alabama | 2007 | 0.622142051 | 0.13977186 |
| State_Alabama | 2008 | 0.753776064 | 0.154243156 |
| State_Alabama | 2009 | 0.816090924 | 0.162199113 |
| State_Alabama | 2010 | 0.740836323 | 0.146600392 |
| State_Alabama | 2011 | 0.869502211 | 0.15773375 |
| State_Alabama | 2012 | 0.830499669 | 0.15522629 |
| State_Alabama | 2013 | 0.722327577 | 0.138407281 |
| State_Alabama | 2014 | 1.088250908 | 0.172618019 |
| State_Alabama | 2015 | 0.806939741 | 0.146468528 |
| State_Alabama | 2016 | 0.567474814 | 0.122433168 |
| State_Alabama | 2017 | 0.667799024 | 0.129920776 |
| State_Alabama | 2018 | 0.732793756 | 0.13600494 |
| State_Alabama | 2019 | 0.822993992 | 0.1422516 |
| State_Alabama | 2020 | 0.881533532 | 0.147201591 |
| State_Alabama | 2021 | 1.034504552 | 0.158425906 |
| State_Alabama | 2022 | 0.852166624 | 0.143147805 |
| State_Alabama | 2023 | 1.119167775 | 0.158672573 |
| State_Alabama | 2024 | 0.76821796 | 0.131473864 |
| State_Alaska | 1999 | NA | NA |
| State_Alaska | 2000 | NA | NA |
| State_Alaska | 2001 | NA | NA |
| State_Alaska | 2002 | NA | NA |
| State_Alaska | 2003 | NA | NA |
| State_Alaska | 2004 | NA | NA |
| State_Alaska | 2005 | NA | 0 |
| State_Alaska | 2006 | NA | NA |
| State_Alaska | 2007 | NA | NA |
| State_Alaska | 2008 | NA | NA |
| State_Alaska | 2009 | NA | NA |
| State_Alaska | 2010 | NA | NA |
| State_Alaska | 2011 | NA | NA |
| State_Alaska | 2012 | NA | NA |
| State_Alaska | 2013 | NA | NA |
| State_Alaska | 2014 | NA | NA |
| State_Alaska | 2015 | NA | NA |
| State_Alaska | 2016 | NA | NA |
| State_Alaska | 2017 | NA | NA |
| State_Alaska | 2018 | NA | NA |
| State_Alaska | 2019 | NA | NA |
| State_Alaska | 2020 | NA | NA |
| State_Alaska | 2021 | NA | NA |
| State_Alaska | 2022 | NA | NA |
| State_Alaska | 2023 | NA | NA |
| State_Alaska | 2024 | NA | NA |
| State_Arizona | 1999 | NA | 0.113276989 |
| State_Arizona | 2000 | NA | 0.126529831 |
| State_Arizona | 2001 | NA | 0.130246055 |
| State_Arizona | 2002 | NA | 0.095340432 |
| State_Arizona | 2003 | 0.693167633 | 0.138855994 |
| State_Arizona | 2004 | NA | 0.102890439 |
| State_Arizona | 2005 | 0.62354832 | 0.127745133 |
| State_Arizona | 2006 | NA | 0.110075543 |
| State_Arizona | 2007 | NA | 0.09808051 |
| State_Arizona | 2008 | NA | 0.097777604 |
| State_Arizona | 2009 | NA | 0.096582941 |
| State_Arizona | 2010 | 0.430030059 | 0.096695629 |
| State_Arizona | 2011 | 0.701881421 | 0.122952582 |
| State_Arizona | 2012 | 0.971080658 | 0.141160624 |
| State_Arizona | 2013 | 0.873191933 | 0.133378039 |
| State_Arizona | 2014 | 0.796454086 | 0.123616813 |
| State_Arizona | 2015 | 0.785657253 | 0.119283679 |
| State_Arizona | 2016 | 0.897532386 | 0.12559659 |
| State_Arizona | 2017 | 0.785021483 | 0.119143796 |
| State_Arizona | 2018 | 0.840282249 | 0.117185348 |
| State_Arizona | 2019 | 0.923216642 | 0.120662151 |
| State_Arizona | 2020 | 1.083177289 | 0.12846439 |
| State_Arizona | 2021 | 1.040614135 | 0.129347026 |
| State_Arizona | 2022 | 0.96200741 | 0.121638813 |
| State_Arizona | 2023 | 1.238098353 | 0.135366303 |
| State_Arizona | 2024 | 0.985505641 | 0.11835925 |
| State_Arkansas | 1999 | NA | NA |
| State_Arkansas | 2000 | NA | NA |
| State_Arkansas | 2001 | NA | 0.170242721 |
| State_Arkansas | 2002 | NA | 0.169581056 |
| State_Arkansas | 2003 | NA | 0.197349916 |
| State_Arkansas | 2004 | NA | 0.165051355 |
| State_Arkansas | 2005 | 1.107951605 | 0.236760734 |
| State_Arkansas | 2006 | NA | 0.203251463 |
| State_Arkansas | 2007 | NA | 0.187956786 |
| State_Arkansas | 2008 | 0.951227518 | 0.213716789 |
| State_Arkansas | 2009 | 1.198715279 | 0.240460867 |
| State_Arkansas | 2010 | 1.469680112 | 0.265468272 |
| State_Arkansas | 2011 | 0.876925948 | 0.197384025 |
| State_Arkansas | 2012 | 1.117500869 | 0.225249745 |
| State_Arkansas | 2013 | 1.18069162 | 0.239041835 |
| State_Arkansas | 2014 | 1.207313049 | 0.230640814 |
| State_Arkansas | 2015 | NA | 0.188994837 |
| State_Arkansas | 2016 | 1.197810223 | 0.230296963 |
| State_Arkansas | 2017 | 1.600881949 | 0.2609661 |
| State_Arkansas | 2018 | 1.210984643 | 0.223685353 |
| State_Arkansas | 2019 | 1.447772149 | 0.236420534 |
| State_Arkansas | 2020 | 1.176596692 | 0.209028807 |
| State_Arkansas | 2021 | 2.121702482 | 0.296248025 |
| State_Arkansas | 2022 | 2.011283142 | 0.277749368 |
| State_Arkansas | 2023 | 2.089761466 | 0.282789464 |
| State_Arkansas | 2024 | 1.522564983 | 0.240839117 |
| State_California | 1999 | 1.06588855 | 0.076548675 |
| State_California | 2000 | 1.073044365 | 0.076288161 |
| State_California | 2001 | 1.181084991 | 0.07909283 |
| State_California | 2002 | 1.233847239 | 0.080784313 |
| State_California | 2003 | 1.203060827 | 0.077722616 |
| State_California | 2004 | 1.416999713 | 0.084346674 |
| State_California | 2005 | 1.314185835 | 0.080375248 |
| State_California | 2006 | 1.420269355 | 0.083513132 |
| State_California | 2007 | 1.453364155 | 0.083148307 |
| State_California | 2008 | 1.462633098 | 0.082323091 |
| State_California | 2009 | 1.202781062 | 0.07383895 |
| State_California | 2010 | 1.528910293 | 0.082774507 |
| State_California | 2011 | 1.328075257 | 0.075671013 |
| State_California | 2012 | 1.527534573 | 0.080379134 |
| State_California | 2013 | 1.507991915 | 0.078843269 |
| State_California | 2014 | 1.333486524 | 0.072373286 |
| State_California | 2015 | 1.298787017 | 0.07135045 |
| State_California | 2016 | 1.35679826 | 0.071851232 |
| State_California | 2017 | 1.3501778 | 0.070543265 |
| State_California | 2018 | 1.325913597 | 0.068756154 |
| State_California | 2019 | 1.273454594 | 0.067278033 |
| State_California | 2020 | 1.464265063 | 0.071802685 |
| State_California | 2021 | 1.513527514 | 0.073523178 |
| State_California | 2022 | 1.39770838 | 0.068998802 |
| State_California | 2023 | 1.36576184 | 0.066821219 |
| State_California | 2024 | 1.346682023 | 0.0659309 |
| State_Colorado | 1999 | NA | 0.192530314 |
| State_Colorado | 2000 | NA | 0.177015698 |
| State_Colorado | 2001 | NA | 0.199466879 |
| State_Colorado | 2002 | 0.930851755 | 0.203825308 |
| State_Colorado | 2003 | NA | 0.170567104 |
| State_Colorado | 2004 | 0.980117069 | 0.202879224 |
| State_Colorado | 2005 | 1.231361114 | 0.226393646 |
| State_Colorado | 2006 | 1.138316948 | 0.215695897 |
| State_Colorado | 2007 | 1.081691957 | 0.20287708 |
| State_Colorado | 2008 | 1.243184145 | 0.217634549 |
| State_Colorado | 2009 | 0.973327374 | 0.187086201 |
| State_Colorado | 2010 | 0.939052402 | 0.186375029 |
| State_Colorado | 2011 | 0.745622657 | 0.159446635 |
| State_Colorado | 2012 | 0.67311735 | 0.145060058 |
| State_Colorado | 2013 | 0.868748998 | 0.167212852 |
| State_Colorado | 2014 | 1.167490098 | 0.193192161 |
| State_Colorado | 2015 | 1.670907496 | 0.223435452 |
| State_Colorado | 2016 | 1.890285902 | 0.237334013 |
| State_Colorado | 2017 | 2.051094712 | 0.243254616 |
| State_Colorado | 2018 | 1.433292781 | 0.198831892 |
| State_Colorado | 2019 | 1.951980759 | 0.228993653 |
| State_Colorado | 2020 | 2.031601862 | 0.224128722 |
| State_Colorado | 2021 | 2.804088002 | 0.268336716 |
| State_Colorado | 2022 | 1.949029479 | 0.218789719 |
| State_Colorado | 2023 | 2.311038403 | 0.234118527 |
| State_Colorado | 2024 | 1.62710006 | 0.193564013 |
| State_Connecticut | 1999 | NA | 0.170674515 |
| State_Connecticut | 2000 | 0.831753602 | 0.186384016 |
| State_Connecticut | 2001 | 0.912473451 | 0.195192101 |
| State_Connecticut | 2002 | 1.469297234 | 0.245688511 |
| State_Connecticut | 2003 | 1.40655018 | 0.238966021 |
| State_Connecticut | 2004 | 1.113035607 | 0.207711616 |
| State_Connecticut | 2005 | 0.95592656 | 0.192499504 |
| State_Connecticut | 2006 | 1.263683512 | 0.225627436 |
| State_Connecticut | 2007 | 0.875206213 | 0.185022161 |
| State_Connecticut | 2008 | 1.070353267 | 0.203993804 |
| State_Connecticut | 2009 | 1.28559468 | 0.219846651 |
| State_Connecticut | 2010 | 1.067282009 | 0.203978742 |
| State_Connecticut | 2011 | 0.920110157 | 0.186617095 |
| State_Connecticut | 2012 | 1.023425293 | 0.19054894 |
| State_Connecticut | 2013 | 0.826873642 | 0.172648135 |
| State_Connecticut | 2014 | 0.764966177 | 0.170064007 |
| State_Connecticut | 2015 | 1.015858269 | 0.189587644 |
| State_Connecticut | 2016 | 0.748210511 | 0.159935514 |
| State_Connecticut | 2017 | NA | 0.136234692 |
| State_Connecticut | 2018 | NA | 0.130833845 |
| State_Connecticut | 2019 | 0.812327192 | 0.15829572 |
| State_Connecticut | 2020 | 0.908102088 | 0.167823514 |
| State_Connecticut | 2021 | 0.865106193 | 0.168081122 |
| State_Connecticut | 2022 | 0.758568633 | 0.155288096 |
| State_Connecticut | 2023 | 0.612405977 | 0.134758638 |
| State_Connecticut | 2024 | 0.798937697 | 0.152067662 |
| State_Delaware | 1999 | NA | NA |
| State_Delaware | 2000 | NA | NA |
| State_Delaware | 2001 | NA | NA |
| State_Delaware | 2002 | NA | NA |
| State_Delaware | 2003 | NA | NA |
| State_Delaware | 2004 | NA | NA |
| State_Delaware | 2005 | NA | NA |
| State_Delaware | 2006 | NA | 0.526142237 |
| State_Delaware | 2007 | NA | 0.579684777 |
| State_Delaware | 2008 | NA | NA |
| State_Delaware | 2009 | NA | NA |
| State_Delaware | 2010 | NA | NA |
| State_Delaware | 2011 | NA | NA |
| State_Delaware | 2012 | NA | NA |
| State_Delaware | 2013 | NA | NA |
| State_Delaware | 2014 | NA | NA |
| State_Delaware | 2015 | NA | NA |
| State_Delaware | 2016 | NA | NA |
| State_Delaware | 2017 | NA | NA |
| State_Delaware | 2018 | NA | NA |
| State_Delaware | 2019 | NA | NA |
| State_Delaware | 2020 | NA | 0.397089167 |
| State_Delaware | 2021 | 1.7840045 | 0.452231912 |
| State_Delaware | 2022 | 2.672377938 | 0.563438159 |
| State_Delaware | 2023 | 2.859912017 | 0.54701903 |
| State_Delaware | 2024 | 2.958266922 | 0.557564602 |
| State_District of Columbia | 1999 | NA | NA |
| State_District of Columbia | 2000 | NA | NA |
| State_District of Columbia | 2001 | NA | NA |
| State_District of Columbia | 2002 | NA | NA |
| State_District of Columbia | 2003 | NA | NA |
| State_District of Columbia | 2004 | NA | NA |
| State_District of Columbia | 2005 | NA | NA |
| State_District of Columbia | 2006 | NA | NA |
| State_District of Columbia | 2007 | NA | NA |
| State_District of Columbia | 2008 | NA | NA |
| State_District of Columbia | 2009 | NA | NA |
| State_District of Columbia | 2010 | NA | NA |
| State_District of Columbia | 2011 | NA | NA |
| State_District of Columbia | 2012 | NA | NA |
| State_District of Columbia | 2013 | NA | 0 |
| State_District of Columbia | 2014 | NA | NA |
| State_District of Columbia | 2015 | NA | NA |
| State_District of Columbia | 2016 | NA | NA |
| State_District of Columbia | 2017 | NA | NA |
| State_District of Columbia | 2018 | NA | NA |
| State_District of Columbia | 2019 | NA | 0 |
| State_District of Columbia | 2020 | NA | NA |
| State_District of Columbia | 2021 | NA | NA |
| State_District of Columbia | 2022 | NA | NA |
| State_District of Columbia | 2023 | NA | NA |
| State_District of Columbia | 2024 | NA | NA |
| State_Florida | 1999 | 0.424586533 | 0.055385838 |
| State_Florida | 2000 | 0.510130422 | 0.060278485 |
| State_Florida | 2001 | 0.49861586 | 0.058968076 |
| State_Florida | 2002 | 0.576477297 | 0.063135712 |
| State_Florida | 2003 | 0.629440356 | 0.065518387 |
| State_Florida | 2004 | 0.577898662 | 0.061630586 |
| State_Florida | 2005 | 0.518251858 | 0.057263454 |
| State_Florida | 2006 | 0.661497112 | 0.065441891 |
| State_Florida | 2007 | 0.702820076 | 0.066413392 |
| State_Florida | 2008 | 0.714930012 | 0.066695237 |
| State_Florida | 2009 | 0.619434879 | 0.061384416 |
| State_Florida | 2010 | 0.609425574 | 0.062036629 |
| State_Florida | 2011 | 0.572399083 | 0.057680374 |
| State_Florida | 2012 | 0.583294918 | 0.056581041 |
| State_Florida | 2013 | 0.691537092 | 0.062451439 |
| State_Florida | 2014 | 0.763523395 | 0.063707324 |
| State_Florida | 2015 | 0.705024097 | 0.059867384 |
| State_Florida | 2016 | 0.832780334 | 0.063606032 |
| State_Florida | 2017 | 0.809224114 | 0.062697596 |
| State_Florida | 2018 | 0.970197178 | 0.066989791 |
| State_Florida | 2019 | 0.941597914 | 0.06487213 |
| State_Florida | 2020 | 1.099753931 | 0.068513607 |
| State_Florida | 2021 | 1.00500503 | 0.068147731 |
| State_Florida | 2022 | 1.12096374 | 0.070165639 |
| State_Florida | 2023 | 1.008190893 | 0.064453847 |
| State_Florida | 2024 | 1.073562836 | 0.065591442 |
| State_Georgia | 1999 | 0.628458424 | 0.126395549 |
| State_Georgia | 2000 | 0.630394257 | 0.124403847 |
| State_Georgia | 2001 | 0.690372943 | 0.131006839 |
| State_Georgia | 2002 | 0.671706938 | 0.127393973 |
| State_Georgia | 2003 | 0.611301775 | 0.118966974 |
| State_Georgia | 2004 | 0.748606712 | 0.131555442 |
| State_Georgia | 2005 | 0.907650951 | 0.143097558 |
| State_Georgia | 2006 | 0.625008081 | 0.117276319 |
| State_Georgia | 2007 | 0.625609759 | 0.115763646 |
| State_Georgia | 2008 | 0.927137861 | 0.136288815 |
| State_Georgia | 2009 | 0.703113885 | 0.117606009 |
| State_Georgia | 2010 | 0.863448627 | 0.130934842 |
| State_Georgia | 2011 | 0.75491158 | 0.121260896 |
| State_Georgia | 2012 | 0.662057205 | 0.109892946 |
| State_Georgia | 2013 | 0.73123814 | 0.112497515 |
| State_Georgia | 2014 | 0.654491617 | 0.105385995 |
| State_Georgia | 2015 | 0.402920139 | 0.084865745 |
| State_Georgia | 2016 | 0.660733409 | 0.102939529 |
| State_Georgia | 2017 | 0.963330196 | 0.122315074 |
| State_Georgia | 2018 | 0.643164929 | 0.097383939 |
| State_Georgia | 2019 | 0.876656757 | 0.111139738 |
| State_Georgia | 2020 | 0.959366217 | 0.116040869 |
| State_Georgia | 2021 | 1.115653821 | 0.123539524 |
| State_Georgia | 2022 | 1.248202162 | 0.128721892 |
| State_Georgia | 2023 | 1.461424876 | 0.137315903 |
| State_Georgia | 2024 | 1.505124825 | 0.139071637 |
| State_Hawaii | 1999 | NA | NA |
| State_Hawaii | 2000 | NA | 0.525121652 |
| State_Hawaii | 2001 | NA | 0.418467817 |
| State_Hawaii | 2002 | NA | NA |
| State_Hawaii | 2003 | NA | NA |
| State_Hawaii | 2004 | NA | 0.377612562 |
| State_Hawaii | 2005 | NA | NA |
| State_Hawaii | 2006 | NA | 0.368459127 |
| State_Hawaii | 2007 | NA | NA |
| State_Hawaii | 2008 | NA | NA |
| State_Hawaii | 2009 | NA | NA |
| State_Hawaii | 2010 | NA | NA |
| State_Hawaii | 2011 | NA | NA |
| State_Hawaii | 2012 | NA | NA |
| State_Hawaii | 2013 | NA | 0.282504027 |
| State_Hawaii | 2014 | NA | 0.27967171 |
| State_Hawaii | 2015 | NA | 0.247453803 |
| State_Hawaii | 2016 | NA | 0.300860211 |
| State_Hawaii | 2017 | NA | NA |
| State_Hawaii | 2018 | NA | NA |
| State_Hawaii | 2019 | NA | NA |
| State_Hawaii | 2020 | NA | 0.282233289 |
| State_Hawaii | 2021 | 1.078202165 | 0.293899052 |
| State_Hawaii | 2022 | 0.800640544 | 0.252354893 |
| State_Hawaii | 2023 | 1.018604256 | 0.263141797 |
| State_Hawaii | 2024 | 0.689717943 | 0.209714543 |
| State_Idaho | 1999 | NA | NA |
| State_Idaho | 2000 | NA | NA |
| State_Idaho | 2001 | NA | NA |
| State_Idaho | 2002 | NA | 0.461739091 |
| State_Idaho | 2003 | NA | NA |
| State_Idaho | 2004 | NA | NA |
| State_Idaho | 2005 | NA | NA |
| State_Idaho | 2006 | NA | NA |
| State_Idaho | 2007 | NA | 0.409317993 |
| State_Idaho | 2008 | NA | NA |
| State_Idaho | 2009 | NA | NA |
| State_Idaho | 2010 | NA | 0.428304869 |
| State_Idaho | 2011 | NA | 0.352711711 |
| State_Idaho | 2012 | NA | 0.359147248 |
| State_Idaho | 2013 | NA | 0.312333892 |
| State_Idaho | 2014 | NA | 0.338218832 |
| State_Idaho | 2015 | NA | 0.335834899 |
| State_Idaho | 2016 | NA | 0.341972676 |
| State_Idaho | 2017 | NA | 0.321858603 |
| State_Idaho | 2018 | 1.876239072 | 0.379837676 |
| State_Idaho | 2019 | 1.527376216 | 0.329933899 |
| State_Idaho | 2020 | 2.007295956 | 0.377322096 |
| State_Idaho | 2021 | 2.353260215 | 0.410206861 |
| State_Idaho | 2022 | 1.861940202 | 0.356435423 |
| State_Idaho | 2023 | 1.697558326 | 0.338458032 |
| State_Idaho | 2024 | 2.670692079 | 0.417011708 |
| State_Illinois | 1999 | 0.928200816 | 0.109404534 |
| State_Illinois | 2000 | 0.919646487 | 0.108454344 |
| State_Illinois | 2001 | 1.051133839 | 0.116176858 |
| State_Illinois | 2002 | 1.05776335 | 0.116973254 |
| State_Illinois | 2003 | 1.038496975 | 0.11495059 |
| State_Illinois | 2004 | 0.879687057 | 0.106893351 |
| State_Illinois | 2005 | 1.030158784 | 0.114123368 |
| State_Illinois | 2006 | 1.028827315 | 0.114013028 |
| State_Illinois | 2007 | 1.020927486 | 0.11189523 |
| State_Illinois | 2008 | 1.060979095 | 0.112704637 |
| State_Illinois | 2009 | 0.916075869 | 0.104388811 |
| State_Illinois | 2010 | 0.782923172 | 0.09707275 |
| State_Illinois | 2011 | 0.896494305 | 0.102404309 |
| State_Illinois | 2012 | 1.150592938 | 0.116367718 |
| State_Illinois | 2013 | 0.950212755 | 0.106508701 |
| State_Illinois | 2014 | 0.959660257 | 0.103607938 |
| State_Illinois | 2015 | 0.84422048 | 0.096349431 |
| State_Illinois | 2016 | 0.939745696 | 0.100330753 |
| State_Illinois | 2017 | 0.864047757 | 0.094322083 |
| State_Illinois | 2018 | 1.008149528 | 0.101557086 |
| State_Illinois | 2019 | 0.910770302 | 0.095305282 |
| State_Illinois | 2020 | 0.983353291 | 0.098574947 |
| State_Illinois | 2021 | 1.12165277 | 0.108307164 |
| State_Illinois | 2022 | 1.046263628 | 0.099767089 |
| State_Illinois | 2023 | 0.954513478 | 0.096746597 |
| State_Illinois | 2024 | 0.758125168 | 0.084311621 |
| State_Indiana | 1999 | 1.021483726 | 0.163679167 |
| State_Indiana | 2000 | 1.362277095 | 0.187216599 |
| State_Indiana | 2001 | 1.223738427 | 0.176744617 |
| State_Indiana | 2002 | 1.154745378 | 0.170468631 |
| State_Indiana | 2003 | 1.065123669 | 0.162754665 |
| State_Indiana | 2004 | 1.0535515 | 0.162665918 |
| State_Indiana | 2005 | 1.156423421 | 0.169040729 |
| State_Indiana | 2006 | 1.057057983 | 0.161394331 |
| State_Indiana | 2007 | 1.284176507 | 0.173790101 |
| State_Indiana | 2008 | 1.374417114 | 0.179525498 |
| State_Indiana | 2009 | 1.104869505 | 0.160427672 |
| State_Indiana | 2010 | 1.12658033 | 0.160297179 |
| State_Indiana | 2011 | 1.375412599 | 0.176076205 |
| State_Indiana | 2012 | 1.398850879 | 0.176518099 |
| State_Indiana | 2013 | 1.680289439 | 0.189576864 |
| State_Indiana | 2014 | 1.605008851 | 0.186466593 |
| State_Indiana | 2015 | 1.601982881 | 0.182261199 |
| State_Indiana | 2016 | 1.344177506 | 0.169651106 |
| State_Indiana | 2017 | 1.215541446 | 0.153814146 |
| State_Indiana | 2018 | 1.799718473 | 0.187562134 |
| State_Indiana | 2019 | 1.678654181 | 0.178433076 |
| State_Indiana | 2020 | 1.471825685 | 0.166271962 |
| State_Indiana | 2021 | 1.843873974 | 0.191908871 |
| State_Indiana | 2022 | 1.633949576 | 0.172575364 |
| State_Indiana | 2023 | 1.583587347 | 0.171225062 |
| State_Indiana | 2024 | 1.927215627 | 0.183297883 |
| State_Iowa | 1999 | NA | 0.194849624 |
| State_Iowa | 2000 | 1.313517222 | 0.241269502 |
| State_Iowa | 2001 | 1.440002196 | 0.256182999 |
| State_Iowa | 2002 | 1.107812472 | 0.222248419 |
| State_Iowa | 2003 | 1.040818435 | 0.209568587 |
| State_Iowa | 2004 | 1.082820195 | 0.218011563 |
| State_Iowa | 2005 | 1.031704603 | 0.21129713 |
| State_Iowa | 2006 | 1.476557057 | 0.251683557 |
| State_Iowa | 2007 | 1.294494318 | 0.231145841 |
| State_Iowa | 2008 | 1.590594176 | 0.262961984 |
| State_Iowa | 2009 | 1.060373732 | 0.216485186 |
| State_Iowa | 2010 | 1.364027234 | 0.233917929 |
| State_Iowa | 2011 | 1.470927182 | 0.251382925 |
| State_Iowa | 2012 | 1.556599187 | 0.249136324 |
| State_Iowa | 2013 | 1.670085243 | 0.266726612 |
| State_Iowa | 2014 | 1.406904723 | 0.234418968 |
| State_Iowa | 2015 | 1.729016549 | 0.258214378 |
| State_Iowa | 2016 | 1.452873367 | 0.23544509 |
| State_Iowa | 2017 | 1.496524604 | 0.244331469 |
| State_Iowa | 2018 | 1.52065036 | 0.239937187 |
| State_Iowa | 2019 | 1.902234437 | 0.265580697 |
| State_Iowa | 2020 | 2.179902773 | 0.27995534 |
| State_Iowa | 2021 | 2.055332905 | 0.28297095 |
| State_Iowa | 2022 | 2.242396476 | 0.282591271 |
| State_Iowa | 2023 | 2.144108989 | 0.276639115 |
| State_Iowa | 2024 | 2.245679158 | 0.282628772 |
| State_Kansas | 1999 | NA | NA |
| State_Kansas | 2000 | NA | 0.224672543 |
| State_Kansas | 2001 | NA | 0.200277127 |
| State_Kansas | 2002 | 1.180238995 | 0.252651799 |
| State_Kansas | 2003 | 1.362291146 | 0.269265435 |
| State_Kansas | 2004 | 1.514319744 | 0.282422435 |
| State_Kansas | 2005 | NA | 0.200107225 |
| State_Kansas | 2006 | 1.268371198 | 0.255395994 |
| State_Kansas | 2007 | 1.118385116 | 0.240697671 |
| State_Kansas | 2008 | 1.090740577 | 0.239224366 |
| State_Kansas | 2009 | NA | 0.233300057 |
| State_Kansas | 2010 | 0.985359923 | 0.222250458 |
| State_Kansas | 2011 | 0.933808922 | 0.211336653 |
| State_Kansas | 2012 | NA | 0.19608633 |
| State_Kansas | 2013 | NA | 0.180341398 |
| State_Kansas | 2014 | 1.055480953 | 0.227634453 |
| State_Kansas | 2015 | 1.065610022 | 0.230449036 |
| State_Kansas | 2016 | 0.930596092 | 0.201514243 |
| State_Kansas | 2017 | 1.019290019 | 0.216485053 |
| State_Kansas | 2018 | 0.97090181 | 0.210582477 |
| State_Kansas | 2019 | 0.883713785 | 0.190070971 |
| State_Kansas | 2020 | 1.539514904 | 0.26033261 |
| State_Kansas | 2021 | 1.536113052 | 0.25985864 |
| State_Kansas | 2022 | 1.517506571 | 0.25232862 |
| State_Kansas | 2023 | 1.304146458 | 0.228453869 |
| State_Kansas | 2024 | 1.397330225 | 0.23945088 |
| State_Kentucky | 1999 | NA | 0.168573728 |
| State_Kentucky | 2000 | 0.983475885 | 0.19691552 |
| State_Kentucky | 2001 | 0.994332194 | 0.195237375 |
| State_Kentucky | 2002 | 0.892906398 | 0.182705116 |
| State_Kentucky | 2003 | 0.791332299 | 0.169456447 |
| State_Kentucky | 2004 | 1.096883059 | 0.201296435 |
| State_Kentucky | 2005 | 1.028240296 | 0.19482967 |
| State_Kentucky | 2006 | 0.842292356 | 0.177095027 |
| State_Kentucky | 2007 | 0.744955632 | 0.1634385 |
| State_Kentucky | 2008 | 1.185271755 | 0.20458558 |
| State_Kentucky | 2009 | 1.946516275 | 0.259689622 |
| State_Kentucky | 2010 | 1.725688533 | 0.247590148 |
| State_Kentucky | 2011 | 1.954669176 | 0.254898604 |
| State_Kentucky | 2012 | 1.667250611 | 0.232281908 |
| State_Kentucky | 2013 | 1.721518064 | 0.23360878 |
| State_Kentucky | 2014 | 1.420958976 | 0.214353809 |
| State_Kentucky | 2015 | 2.167337704 | 0.257198973 |
| State_Kentucky | 2016 | 2.361873444 | 0.269257551 |
| State_Kentucky | 2017 | 2.462857834 | 0.270133993 |
| State_Kentucky | 2018 | 2.159212069 | 0.247235444 |
| State_Kentucky | 2019 | 2.339982834 | 0.255256246 |
| State_Kentucky | 2020 | 2.530506034 | 0.265554492 |
| State_Kentucky | 2021 | 3.152432071 | 0.300305353 |
| State_Kentucky | 2022 | 2.617811034 | 0.270689564 |
| State_Kentucky | 2023 | 2.793000539 | 0.27625379 |
| State_Kentucky | 2024 | 2.901548478 | 0.275243508 |
| State_Louisiana | 1999 | NA | 0.151986189 |
| State_Louisiana | 2000 | NA | 0.142297691 |
| State_Louisiana | 2001 | NA | 0.136672705 |
| State_Louisiana | 2002 | 0.790471361 | 0.173107939 |
| State_Louisiana | 2003 | NA | 0.154101603 |
| State_Louisiana | 2004 | NA | 0.146874019 |
| State_Louisiana | 2005 | NA | 0.128202283 |
| State_Louisiana | 2006 | NA | 0.139701882 |
| State_Louisiana | 2007 | NA | 0.159510653 |
| State_Louisiana | 2008 | 0.703531691 | 0.158596719 |
| State_Louisiana | 2009 | NA | 0.131802285 |
| State_Louisiana | 2010 | 0.710218737 | 0.159793943 |
| State_Louisiana | 2011 | NA | 0.137998998 |
| State_Louisiana | 2012 | 1.058548187 | 0.191471067 |
| State_Louisiana | 2013 | 0.769778545 | 0.156490986 |
| State_Louisiana | 2014 | 0.783280525 | 0.158696176 |
| State_Louisiana | 2015 | 0.908699892 | 0.171392131 |
| State_Louisiana | 2016 | 1.197892509 | 0.190197366 |
| State_Louisiana | 2017 | 1.344607381 | 0.199252942 |
| State_Louisiana | 2018 | 1.301837674 | 0.195159417 |
| State_Louisiana | 2019 | 1.352596306 | 0.196128632 |
| State_Louisiana | 2020 | 1.359945188 | 0.195070378 |
| State_Louisiana | 2021 | 1.550013929 | 0.212468214 |
| State_Louisiana | 2022 | 1.788396345 | 0.227076906 |
| State_Louisiana | 2023 | 1.453079046 | 0.199547947 |
| State_Louisiana | 2024 | 2.017742897 | 0.234682926 |
| State_Maine | 1999 | NA | 0.391200413 |
| State_Maine | 2000 | NA | 0.445851441 |
| State_Maine | 2001 | NA | 0.395044437 |
| State_Maine | 2002 | NA | 0.423904719 |
| State_Maine | 2003 | NA | NA |
| State_Maine | 2004 | NA | 0.407096812 |
| State_Maine | 2005 | NA | 0.408590645 |
| State_Maine | 2006 | NA | 0.391080716 |
| State_Maine | 2007 | NA | 0.336496133 |
| State_Maine | 2008 | NA | 0.342634509 |
| State_Maine | 2009 | NA | 0.303070916 |
| State_Maine | 2010 | NA | 0.361426588 |
| State_Maine | 2011 | NA | 0.334454116 |
| State_Maine | 2012 | NA | 0.339600091 |
| State_Maine | 2013 | NA | 0.30930843 |
| State_Maine | 2014 | 1.635376702 | 0.369249712 |
| State_Maine | 2015 | NA | 0.319405679 |
| State_Maine | 2016 | NA | 0.283070397 |
| State_Maine | 2017 | NA | 0.337648409 |
| State_Maine | 2018 | NA | 0.3114833 |
| State_Maine | 2019 | 1.65781153 | 0.355265308 |
| State_Maine | 2020 | 1.986527349 | 0.38532835 |
| State_Maine | 2021 | 1.192701116 | 0.295278241 |
| State_Maine | 2022 | 2.400252658 | 0.421212067 |
| State_Maine | 2023 | 1.49400198 | 0.321132119 |
| State_Maine | 2024 | 1.875025656 | 0.349793116 |
| State_Maryland | 1999 | 1.270367035 | 0.203772817 |
| State_Maryland | 2000 | 1.43023995 | 0.216311024 |
| State_Maryland | 2001 | 1.24640493 | 0.197789072 |
| State_Maryland | 2002 | 1.186450367 | 0.192817057 |
| State_Maryland | 2003 | 1.157693931 | 0.186500758 |
| State_Maryland | 2004 | 1.039846927 | 0.176529542 |
| State_Maryland | 2005 | 1.12078656 | 0.182802503 |
| State_Maryland | 2006 | 1.27997013 | 0.194183624 |
| State_Maryland | 2007 | 1.263733062 | 0.189670446 |
| State_Maryland | 2008 | 1.21797747 | 0.186505266 |
| State_Maryland | 2009 | 1.619914777 | 0.213251338 |
| State_Maryland | 2010 | 1.177372033 | 0.177252496 |
| State_Maryland | 2011 | 1.294608183 | 0.183813456 |
| State_Maryland | 2012 | 1.386475755 | 0.189666864 |
| State_Maryland | 2013 | 1.116247409 | 0.169771526 |
| State_Maryland | 2014 | 0.904482339 | 0.155517948 |
| State_Maryland | 2015 | 0.740953774 | 0.133354317 |
| State_Maryland | 2016 | 0.64579877 | 0.126116375 |
| State_Maryland | 2017 | 1.199938333 | 0.164229812 |
| State_Maryland | 2018 | 1.249140493 | 0.166023061 |
| State_Maryland | 2019 | 1.437717268 | 0.17894563 |
| State_Maryland | 2020 | 1.809265712 | 0.193662407 |
| State_Maryland | 2021 | 1.501942421 | 0.179706716 |
| State_Maryland | 2022 | 1.998891605 | 0.195272833 |
| State_Maryland | 2023 | 2.499973726 | 0.223141412 |
| State_Maryland | 2024 | 2.094359183 | 0.199210456 |
| State_Massachusetts | 1999 | 1.032894316 | 0.152406978 |
| State_Massachusetts | 2000 | 1.004414058 | 0.149921366 |
| State_Massachusetts | 2001 | 1.091150376 | 0.154599045 |
| State_Massachusetts | 2002 | 1.088446686 | 0.155976709 |
| State_Massachusetts | 2003 | 1.10147681 | 0.156195859 |
| State_Massachusetts | 2004 | 0.9756204 | 0.147620551 |
| State_Massachusetts | 2005 | 0.968863853 | 0.14536216 |
| State_Massachusetts | 2006 | 1.425398715 | 0.176542545 |
| State_Massachusetts | 2007 | 1.000988233 | 0.150262326 |
| State_Massachusetts | 2008 | 1.119869672 | 0.155236351 |
| State_Massachusetts | 2009 | 0.852736111 | 0.135850702 |
| State_Massachusetts | 2010 | 0.966066782 | 0.139714229 |
| State_Massachusetts | 2011 | 0.731670582 | 0.123367546 |
| State_Massachusetts | 2012 | 0.829194879 | 0.12680447 |
| State_Massachusetts | 2013 | 0.877906304 | 0.133079668 |
| State_Massachusetts | 2014 | 0.890421328 | 0.130523689 |
| State_Massachusetts | 2015 | 0.820611411 | 0.127718088 |
| State_Massachusetts | 2016 | 0.908076793 | 0.131292727 |
| State_Massachusetts | 2017 | 0.74220325 | 0.113783279 |
| State_Massachusetts | 2018 | 0.707362733 | 0.110808499 |
| State_Massachusetts | 2019 | 0.779403971 | 0.115295368 |
| State_Massachusetts | 2020 | 0.977936038 | 0.133248004 |
| State_Massachusetts | 2021 | 0.947410339 | 0.127826084 |
| State_Massachusetts | 2022 | 0.735805998 | 0.113186991 |
| State_Massachusetts | 2023 | 1.093261563 | 0.134110688 |
| State_Massachusetts | 2024 | 1.060662289 | 0.132549709 |
| State_Michigan | 1999 | 1.23897359 | 0.143146684 |
| State_Michigan | 2000 | 0.842527228 | 0.116978796 |
| State_Michigan | 2001 | 1.400453389 | 0.149393383 |
| State_Michigan | 2002 | 1.114141211 | 0.132395034 |
| State_Michigan | 2003 | 1.234144674 | 0.137546384 |
| State_Michigan | 2004 | 0.875743629 | 0.117519492 |
| State_Michigan | 2005 | 1.243856802 | 0.136928124 |
| State_Michigan | 2006 | 1.159510799 | 0.131862967 |
| State_Michigan | 2007 | 1.124139111 | 0.127860002 |
| State_Michigan | 2008 | 1.237893061 | 0.133495168 |
| State_Michigan | 2009 | 1.146129044 | 0.128059111 |
| State_Michigan | 2010 | 1.332230497 | 0.135810272 |
| State_Michigan | 2011 | 1.210267296 | 0.128789894 |
| State_Michigan | 2012 | 1.275299406 | 0.134176977 |
| State_Michigan | 2013 | 1.370280839 | 0.135388605 |
| State_Michigan | 2014 | 1.322093021 | 0.132037489 |
| State_Michigan | 2015 | 1.098984937 | 0.119603456 |
| State_Michigan | 2016 | 1.203523734 | 0.122349474 |
| State_Michigan | 2017 | 1.043442985 | 0.115516635 |
| State_Michigan | 2018 | 1.011036959 | 0.112477982 |
| State_Michigan | 2019 | 0.970514728 | 0.107237953 |
| State_Michigan | 2020 | 1.404830146 | 0.127712259 |
| State_Michigan | 2021 | 1.511999532 | 0.136785457 |
| State_Michigan | 2022 | 1.316676296 | 0.124606507 |
| State_Michigan | 2023 | 1.017314121 | 0.107473432 |
| State_Michigan | 2024 | 1.003131781 | 0.105561145 |
| State_Minnesota | 1999 | 0.996032492 | 0.179798473 |
| State_Minnesota | 2000 | 1.239752968 | 0.199009029 |
| State_Minnesota | 2001 | 1.2271039 | 0.197551475 |
| State_Minnesota | 2002 | 1.510219473 | 0.214710213 |
| State_Minnesota | 2003 | 1.666465179 | 0.227884355 |
| State_Minnesota | 2004 | 1.474910686 | 0.211811212 |
| State_Minnesota | 2005 | 1.541735581 | 0.215221623 |
| State_Minnesota | 2006 | 1.632660484 | 0.221528993 |
| State_Minnesota | 2007 | 1.395229523 | 0.200988104 |
| State_Minnesota | 2008 | 1.565983356 | 0.211089689 |
| State_Minnesota | 2009 | 1.633745864 | 0.213193481 |
| State_Minnesota | 2010 | 1.873288236 | 0.23111611 |
| State_Minnesota | 2011 | 1.665144684 | 0.211099848 |
| State_Minnesota | 2012 | 1.887163408 | 0.225628755 |
| State_Minnesota | 2013 | 2.117469005 | 0.23444507 |
| State_Minnesota | 2014 | 1.581054794 | 0.197645611 |
| State_Minnesota | 2015 | 2.237785788 | 0.233125345 |
| State_Minnesota | 2016 | 1.953275795 | 0.217619618 |
| State_Minnesota | 2017 | 2.034502209 | 0.217679093 |
| State_Minnesota | 2018 | 2.424680118 | 0.237314458 |
| State_Minnesota | 2019 | 2.140633582 | 0.217760122 |
| State_Minnesota | 2020 | 2.247073225 | 0.221671527 |
| State_Minnesota | 2021 | 2.811656128 | 0.250689961 |
| State_Minnesota | 2022 | 2.830600169 | 0.246710136 |
| State_Minnesota | 2023 | 2.561289279 | 0.231083293 |
| State_Minnesota | 2024 | 2.287777063 | 0.214643466 |
| State_Mississippi | 1999 | NA | 0.224563724 |
| State_Mississippi | 2000 | NA | 0.204155417 |
| State_Mississippi | 2001 | NA | 0.21051529 |
| State_Mississippi | 2002 | 1.170240881 | 0.25582046 |
| State_Mississippi | 2003 | NA | 0.182465316 |
| State_Mississippi | 2004 | 1.259085159 | 0.263753903 |
| State_Mississippi | 2005 | NA | 0.237569655 |
| State_Mississippi | 2006 | NA | 0.230103704 |
| State_Mississippi | 2007 | 1.369505301 | 0.270162957 |
| State_Mississippi | 2008 | NA | 0.209083303 |
| State_Mississippi | 2009 | NA | 0.20825313 |
| State_Mississippi | 2010 | 1.197470207 | 0.247262585 |
| State_Mississippi | 2011 | 1.473536536 | 0.271500608 |
| State_Mississippi | 2012 | 1.391626626 | 0.260594075 |
| State_Mississippi | 2013 | 1.131579526 | 0.228279259 |
| State_Mississippi | 2014 | 1.271933294 | 0.248577504 |
| State_Mississippi | 2015 | 1.529729961 | 0.265657853 |
| State_Mississippi | 2016 | 1.769426151 | 0.28254498 |
| State_Mississippi | 2017 | 1.739753828 | 0.278878013 |
| State_Mississippi | 2018 | 1.791109532 | 0.282867512 |
| State_Mississippi | 2019 | 1.806679541 | 0.278965268 |
| State_Mississippi | 2020 | 2.298158239 | 0.31072838 |
| State_Mississippi | 2021 | 2.31871265 | 0.320259408 |
| State_Mississippi | 2022 | 2.512815182 | 0.325670993 |
| State_Mississippi | 2023 | 2.752628452 | 0.340809395 |
| State_Mississippi | 2024 | 2.159541489 | 0.296606239 |
| State_Missouri | 1999 | 0.745162508 | 0.138515015 |
| State_Missouri | 2000 | 1.294756152 | 0.183201153 |
| State_Missouri | 2001 | 1.093180647 | 0.168785097 |
| State_Missouri | 2002 | 1.062644489 | 0.164085268 |
| State_Missouri | 2003 | 0.927119647 | 0.152506364 |
| State_Missouri | 2004 | 1.072808193 | 0.163771933 |
| State_Missouri | 2005 | 1.036734592 | 0.160243489 |
| State_Missouri | 2006 | 0.941939281 | 0.149473596 |
| State_Missouri | 2007 | 1.16442744 | 0.168472414 |
| State_Missouri | 2008 | 1.336353635 | 0.176139957 |
| State_Missouri | 2009 | 1.28010932 | 0.171774858 |
| State_Missouri | 2010 | 1.352157696 | 0.175574221 |
| State_Missouri | 2011 | 1.148307736 | 0.159860287 |
| State_Missouri | 2012 | 0.987159212 | 0.148224862 |
| State_Missouri | 2013 | 1.02451238 | 0.150829812 |
| State_Missouri | 2014 | 1.031935423 | 0.148823071 |
| State_Missouri | 2015 | 1.31491695 | 0.164654786 |
| State_Missouri | 2016 | 1.092837544 | 0.151537433 |
| State_Missouri | 2017 | 1.449848798 | 0.170194429 |
| State_Missouri | 2018 | 1.033866817 | 0.14214083 |
| State_Missouri | 2019 | 0.919377416 | 0.133819644 |
| State_Missouri | 2020 | 1.022385306 | 0.140112378 |
| State_Missouri | 2021 | 1.301036397 | 0.161181426 |
| State_Missouri | 2022 | 1.394978696 | 0.160840999 |
| State_Missouri | 2023 | 1.305459363 | 0.156585811 |
| State_Missouri | 2024 | 1.626307116 | 0.170658766 |
| State_Montana | 1999 | NA | NA |
| State_Montana | 2000 | NA | NA |
| State_Montana | 2001 | NA | NA |
| State_Montana | 2002 | NA | NA |
| State_Montana | 2003 | NA | NA |
| State_Montana | 2004 | NA | NA |
| State_Montana | 2005 | NA | NA |
| State_Montana | 2006 | NA | NA |
| State_Montana | 2007 | NA | NA |
| State_Montana | 2008 | NA | NA |
| State_Montana | 2009 | NA | 0.462700405 |
| State_Montana | 2010 | NA | NA |
| State_Montana | 2011 | NA | NA |
| State_Montana | 2012 | NA | 0.432212691 |
| State_Montana | 2013 | NA | NA |
| State_Montana | 2014 | NA | NA |
| State_Montana | 2015 | NA | NA |
| State_Montana | 2016 | NA | NA |
| State_Montana | 2017 | NA | 0.365061245 |
| State_Montana | 2018 | NA | NA |
| State_Montana | 2019 | NA | 0.407176621 |
| State_Montana | 2020 | NA | NA |
| State_Montana | 2021 | 1.864476366 | 0.445757396 |
| State_Montana | 2022 | 2.628688054 | 0.516105948 |
| State_Montana | 2023 | 1.753126217 | 0.417954141 |
| State_Montana | 2024 | 1.991620012 | 0.428818266 |
| State_Nebraska | 1999 | 1.644330119 | 0.368993322 |
| State_Nebraska | 2000 | NA | 0.351907588 |
| State_Nebraska | 2001 | NA | 0.319839861 |
| State_Nebraska | 2002 | NA | 0.353503004 |
| State_Nebraska | 2003 | NA | 0.247994267 |
| State_Nebraska | 2004 | NA | 0.336449277 |
| State_Nebraska | 2005 | 1.89997979 | 0.389694224 |
| State_Nebraska | 2006 | 1.912208662 | 0.399477951 |
| State_Nebraska | 2007 | 1.698770357 | 0.365223851 |
| State_Nebraska | 2008 | 1.727223838 | 0.362760876 |
| State_Nebraska | 2009 | 1.758653658 | 0.369404826 |
| State_Nebraska | 2010 | 1.911378427 | 0.384957308 |
| State_Nebraska | 2011 | 2.565500183 | 0.43863997 |
| State_Nebraska | 2012 | 2.130564544 | 0.411666462 |
| State_Nebraska | 2013 | NA | 0.281628403 |
| State_Nebraska | 2014 | 1.750676337 | 0.354092986 |
| State_Nebraska | 2015 | 1.876763119 | 0.366012274 |
| State_Nebraska | 2016 | 2.125864879 | 0.386970793 |
| State_Nebraska | 2017 | 2.853111819 | 0.44126086 |
| State_Nebraska | 2018 | 1.702437789 | 0.345188927 |
| State_Nebraska | 2019 | 2.235478008 | 0.376708041 |
| State_Nebraska | 2020 | 2.380534761 | 0.390574938 |
| State_Nebraska | 2021 | 3.901796283 | 0.51061193 |
| State_Nebraska | 2022 | 3.184957547 | 0.445540189 |
| State_Nebraska | 2023 | 2.08502491 | 0.369331145 |
| State_Nebraska | 2024 | 3.432095415 | 0.45912605 |
| State_Nevada | 1999 | NA | NA |
| State_Nevada | 2000 | NA | NA |
| State_Nevada | 2001 | NA | NA |
| State_Nevada | 2002 | NA | NA |
| State_Nevada | 2003 | NA | NA |
| State_Nevada | 2004 | NA | NA |
| State_Nevada | 2005 | NA | 0.232745966 |
| State_Nevada | 2006 | NA | NA |
| State_Nevada | 2007 | NA | NA |
| State_Nevada | 2008 | NA | NA |
| State_Nevada | 2009 | NA | NA |
| State_Nevada | 2010 | NA | NA |
| State_Nevada | 2011 | NA | NA |
| State_Nevada | 2012 | NA | NA |
| State_Nevada | 2013 | NA | NA |
| State_Nevada | 2014 | NA | 0.237562917 |
| State_Nevada | 2015 | NA | NA |
| State_Nevada | 2016 | NA | 0.162976987 |
| State_Nevada | 2017 | NA | 0.172691338 |
| State_Nevada | 2018 | NA | 0.151920595 |
| State_Nevada | 2019 | NA | 0.185388687 |
| State_Nevada | 2020 | NA | 0.171290502 |
| State_Nevada | 2021 | 1.60382296 | 0.267746104 |
| State_Nevada | 2022 | 1.546970259 | 0.251850554 |
| State_Nevada | 2023 | 1.121778064 | 0.209908468 |
| State_Nevada | 2024 | 1.004666782 | 0.199462832 |
| State_New Hampshire | 1999 | NA | 0.498673136 |
| State_New Hampshire | 2000 | NA | NA |
| State_New Hampshire | 2001 | NA | NA |
| State_New Hampshire | 2002 | NA | 0.460840505 |
| State_New Hampshire | 2003 | NA | NA |
| State_New Hampshire | 2004 | NA | 0.409318672 |
| State_New Hampshire | 2005 | NA | NA |
| State_New Hampshire | 2006 | NA | 0.497341117 |
| State_New Hampshire | 2007 | NA | NA |
| State_New Hampshire | 2008 | NA | 0.331980362 |
| State_New Hampshire | 2009 | NA | 0.388071209 |
| State_New Hampshire | 2010 | 2.119544241 | 0.478115176 |
| State_New Hampshire | 2011 | NA | 0.347762712 |
| State_New Hampshire | 2012 | NA | 0.434801488 |
| State_New Hampshire | 2013 | NA | 0.345946991 |
| State_New Hampshire | 2014 | 1.977834243 | 0.438185394 |
| State_New Hampshire | 2015 | NA | 0.371013328 |
| State_New Hampshire | 2016 | NA | 0.353414651 |
| State_New Hampshire | 2017 | NA | 0.355787099 |
| State_New Hampshire | 2018 | NA | 0.313897355 |
| State_New Hampshire | 2019 | 1.7603807 | 0.389755716 |
| State_New Hampshire | 2020 | NA | 0.354086177 |
| State_New Hampshire | 2021 | 1.403595201 | 0.353242963 |
| State_New Hampshire | 2022 | 1.396559812 | 0.329972846 |
| State_New Hampshire | 2023 | 1.186758167 | 0.292846136 |
| State_New Hampshire | 2024 | 0.70897558 | 0.225070462 |
| State_New Jersey | 1999 | 0.674852433 | 0.109643207 |
| State_New Jersey | 2000 | 0.869111741 | 0.124398218 |
| State_New Jersey | 2001 | 0.888866322 | 0.12454364 |
| State_New Jersey | 2002 | 0.989479504 | 0.131222102 |
| State_New Jersey | 2003 | 0.794975466 | 0.11603054 |
| State_New Jersey | 2004 | 0.862948851 | 0.122327875 |
| State_New Jersey | 2005 | 0.99925124 | 0.130368494 |
| State_New Jersey | 2006 | 1.067741524 | 0.135241727 |
| State_New Jersey | 2007 | 0.734931451 | 0.108772005 |
| State_New Jersey | 2008 | 0.870463227 | 0.120191558 |
| State_New Jersey | 2009 | 0.924195668 | 0.122142842 |
| State_New Jersey | 2010 | 1.255982059 | 0.140741819 |
| State_New Jersey | 2011 | 1.504288654 | 0.152769254 |
| State_New Jersey | 2012 | 1.055924335 | 0.126149959 |
| State_New Jersey | 2013 | 1.101740734 | 0.128015496 |
| State_New Jersey | 2014 | 1.421384173 | 0.145562716 |
| State_New Jersey | 2015 | 1.065429176 | 0.126657546 |
| State_New Jersey | 2016 | 1.171051167 | 0.130205747 |
| State_New Jersey | 2017 | 1.177193449 | 0.128077438 |
| State_New Jersey | 2018 | 1.016994342 | 0.119128545 |
| State_New Jersey | 2019 | 0.883280555 | 0.109340213 |
| State_New Jersey | 2020 | 0.982828959 | 0.113982847 |
| State_New Jersey | 2021 | 1.127746134 | 0.123064565 |
| State_New Jersey | 2022 | 1.074707633 | 0.118493965 |
| State_New Jersey | 2023 | 1.035504145 | 0.114188973 |
| State_New Jersey | 2024 | 1.220034168 | 0.121440779 |
| State_New Mexico | 1999 | NA | NA |
| State_New Mexico | 2000 | NA | NA |
| State_New Mexico | 2001 | NA | 0.316311228 |
| State_New Mexico | 2002 | 1.727464739 | 0.387251036 |
| State_New Mexico | 2003 | NA | 0.318594753 |
| State_New Mexico | 2004 | NA | NA |
| State_New Mexico | 2005 | NA | NA |
| State_New Mexico | 2006 | NA | 0.244884319 |
| State_New Mexico | 2007 | NA | 0.303226612 |
| State_New Mexico | 2008 | NA | 0.267345952 |
| State_New Mexico | 2009 | NA | 0.31208455 |
| State_New Mexico | 2010 | NA | 0.286142624 |
| State_New Mexico | 2011 | 1.480302479 | 0.3190884 |
| State_New Mexico | 2012 | 1.291067731 | 0.291910878 |
| State_New Mexico | 2013 | NA | 0.252295117 |
| State_New Mexico | 2014 | NA | 0.288245927 |
| State_New Mexico | 2015 | NA | 0.241933277 |
| State_New Mexico | 2016 | NA | 0.264420947 |
| State_New Mexico | 2017 | NA | 0.237917738 |
| State_New Mexico | 2018 | 1.611329581 | 0.320058936 |
| State_New Mexico | 2019 | 1.381510705 | 0.280007465 |
| State_New Mexico | 2020 | 1.338837617 | 0.266460898 |
| State_New Mexico | 2021 | 1.158028489 | 0.247447808 |
| State_New Mexico | 2022 | 1.583364451 | 0.293186308 |
| State_New Mexico | 2023 | 1.150210528 | 0.253289424 |
| State_New Mexico | 2024 | 1.021180307 | 0.23806029 |
| State_New York | 1999 | 0.745620633 | 0.078304121 |
| State_New York | 2000 | 0.777327526 | 0.079105857 |
| State_New York | 2001 | 0.728065147 | 0.076035381 |
| State_New York | 2002 | 0.682613109 | 0.072445776 |
| State_New York | 2003 | 0.682157904 | 0.072847476 |
| State_New York | 2004 | 0.848990693 | 0.080746543 |
| State_New York | 2005 | 0.61110618 | 0.068046527 |
| State_New York | 2006 | 0.865795576 | 0.08065402 |
| State_New York | 2007 | 0.897087708 | 0.082249486 |
| State_New York | 2008 | 0.771917995 | 0.07553091 |
| State_New York | 2009 | 0.991443442 | 0.085575605 |
| State_New York | 2010 | 0.820176206 | 0.076389637 |
| State_New York | 2011 | 0.806994767 | 0.076731548 |
| State_New York | 2012 | 0.944894469 | 0.081219577 |
| State_New York | 2013 | 0.770612262 | 0.072496655 |
| State_New York | 2014 | 0.776112868 | 0.072986398 |
| State_New York | 2015 | 0.735398935 | 0.07050927 |
| State_New York | 2016 | 0.768063728 | 0.072596448 |
| State_New York | 2017 | 0.824734162 | 0.073928224 |
| State_New York | 2018 | 1.010834467 | 0.079355173 |
| State_New York | 2019 | 0.791171467 | 0.069067527 |
| State_New York | 2020 | 1.15373288 | 0.083980626 |
| State_New York | 2021 | 1.268002739 | 0.08801483 |
| State_New York | 2022 | 1.097513251 | 0.079546144 |
| State_New York | 2023 | 1.157727517 | 0.081666774 |
| State_New York | 2024 | 1.19199349 | 0.081442694 |
| State_North Carolina | 1999 | 0.979436997 | 0.143187581 |
| State_North Carolina | 2000 | 0.949993918 | 0.140361347 |
| State_North Carolina | 2001 | 1.339994554 | 0.164091075 |
| State_North Carolina | 2002 | 1.179047662 | 0.152662078 |
| State_North Carolina | 2003 | 1.103329541 | 0.14518904 |
| State_North Carolina | 2004 | 1.193318518 | 0.149734246 |
| State_North Carolina | 2005 | 1.196403921 | 0.149098509 |
| State_North Carolina | 2006 | 1.415394497 | 0.159325942 |
| State_North Carolina | 2007 | 1.7569222 | 0.174654971 |
| State_North Carolina | 2008 | 1.308704266 | 0.14762981 |
| State_North Carolina | 2009 | 1.709474564 | 0.167645405 |
| State_North Carolina | 2010 | 1.167084619 | 0.13668472 |
| State_North Carolina | 2011 | 1.527233817 | 0.156592187 |
| State_North Carolina | 2012 | 1.4186667 | 0.145903121 |
| State_North Carolina | 2013 | 1.402248023 | 0.142719861 |
| State_North Carolina | 2014 | 0.930590414 | 0.113553674 |
| State_North Carolina | 2015 | 1.013168368 | 0.118425958 |
| State_North Carolina | 2016 | 1.025138152 | 0.118825376 |
| State_North Carolina | 2017 | 0.894297217 | 0.107553587 |
| State_North Carolina | 2018 | 0.948627461 | 0.10987495 |
| State_North Carolina | 2019 | 1.123586947 | 0.116133836 |
| State_North Carolina | 2020 | 1.013989708 | 0.108417373 |
| State_North Carolina | 2021 | 1.161315147 | 0.11919188 |
| State_North Carolina | 2022 | 1.608226314 | 0.136455393 |
| State_North Carolina | 2023 | 2.110440035 | 0.156551058 |
| State_North Carolina | 2024 | 1.728387989 | 0.139413572 |
| State_North Dakota | 1999 | NA | 0.724217482 |
| State_North Dakota | 2000 | NA | NA |
| State_North Dakota | 2001 | NA | NA |
| State_North Dakota | 2002 | NA | NA |
| State_North Dakota | 2003 | NA | NA |
| State_North Dakota | 2004 | NA | NA |
| State_North Dakota | 2005 | NA | NA |
| State_North Dakota | 2006 | NA | NA |
| State_North Dakota | 2007 | NA | 0.720335607 |
| State_North Dakota | 2008 | NA | 0.635371283 |
| State_North Dakota | 2009 | NA | 0.570582686 |
| State_North Dakota | 2010 | NA | NA |
| State_North Dakota | 2011 | NA | 0.582751265 |
| State_North Dakota | 2012 | NA | NA |
| State_North Dakota | 2013 | NA | NA |
| State_North Dakota | 2014 | NA | 0.580572545 |
| State_North Dakota | 2015 | NA | 0.631502714 |
| State_North Dakota | 2016 | NA | NA |
| State_North Dakota | 2017 | NA | NA |
| State_North Dakota | 2018 | NA | NA |
| State_North Dakota | 2019 | NA | 0.573651445 |
| State_North Dakota | 2020 | NA | 0.564401206 |
| State_North Dakota | 2021 | NA | NA |
| State_North Dakota | 2022 | 1.597784785 | 0.513717108 |
| State_North Dakota | 2023 | NA | NA |
| State_North Dakota | 2024 | NA | NA |
| State_Ohio | 1999 | 1.382685056 | 0.135218921 |
| State_Ohio | 2000 | 1.370147288 | 0.133749802 |
| State_Ohio | 2001 | 1.512307764 | 0.139894093 |
| State_Ohio | 2002 | 1.546345795 | 0.141210979 |
| State_Ohio | 2003 | 1.899884418 | 0.155261694 |
| State_Ohio | 2004 | 2.025165459 | 0.159265641 |
| State_Ohio | 2005 | 1.956237364 | 0.156291588 |
| State_Ohio | 2006 | 2.013229803 | 0.157810894 |
| State_Ohio | 2007 | 1.914980349 | 0.153777287 |
| State_Ohio | 2008 | 1.824884865 | 0.148508422 |
| State_Ohio | 2009 | 1.457895252 | 0.132927096 |
| State_Ohio | 2010 | 1.564453884 | 0.135833451 |
| State_Ohio | 2011 | 1.504837368 | 0.132330762 |
| State_Ohio | 2012 | 1.630956322 | 0.136255824 |
| State_Ohio | 2013 | 1.790258712 | 0.142931079 |
| State_Ohio | 2014 | 1.534603879 | 0.128258255 |
| State_Ohio | 2015 | 1.718397774 | 0.139373706 |
| State_Ohio | 2016 | 1.52261219 | 0.127575596 |
| State_Ohio | 2017 | 1.620454325 | 0.129830544 |
| State_Ohio | 2018 | 1.099624225 | 0.107147622 |
| State_Ohio | 2019 | 1.474411617 | 0.120272676 |
| State_Ohio | 2020 | 1.722144642 | 0.129898586 |
| State_Ohio | 2021 | 1.719559567 | 0.133954477 |
| State_Ohio | 2022 | 1.395919899 | 0.117595723 |
| State_Ohio | 2023 | 1.367412496 | 0.118732722 |
| State_Ohio | 2024 | 1.391375847 | 0.115910509 |
| State_Oklahoma | 1999 | NA | 0.168204698 |
| State_Oklahoma | 2000 | 0.907661492 | 0.198127326 |
| State_Oklahoma | 2001 | 1.367624405 | 0.241804526 |
| State_Oklahoma | 2002 | 1.593994406 | 0.262314765 |
| State_Oklahoma | 2003 | 1.143938298 | 0.220188625 |
| State_Oklahoma | 2004 | 1.114363847 | 0.215141731 |
| State_Oklahoma | 2005 | 0.947526868 | 0.197841607 |
| State_Oklahoma | 2006 | 1.546929858 | 0.251400175 |
| State_Oklahoma | 2007 | 0.857016096 | 0.187475619 |
| State_Oklahoma | 2008 | 1.587543459 | 0.251879855 |
| State_Oklahoma | 2009 | 1.519571458 | 0.247364835 |
| State_Oklahoma | 2010 | 1.563264133 | 0.240082445 |
| State_Oklahoma | 2011 | 1.253108913 | 0.216461066 |
| State_Oklahoma | 2012 | 1.903467241 | 0.266318038 |
| State_Oklahoma | 2013 | 1.598304928 | 0.237960619 |
| State_Oklahoma | 2014 | 1.897412293 | 0.258252219 |
| State_Oklahoma | 2015 | 1.792594081 | 0.250803424 |
| State_Oklahoma | 2016 | 2.276516015 | 0.285756555 |
| State_Oklahoma | 2017 | 2.338094145 | 0.28058102 |
| State_Oklahoma | 2018 | 2.589819612 | 0.29114146 |
| State_Oklahoma | 2019 | 2.677305864 | 0.295830426 |
| State_Oklahoma | 2020 | 3.578257892 | 0.344214714 |
| State_Oklahoma | 2021 | 3.669674274 | 0.348093268 |
| State_Oklahoma | 2022 | 4.154268677 | 0.368217419 |
| State_Oklahoma | 2023 | 3.898798594 | 0.352429214 |
| State_Oklahoma | 2024 | 3.327734672 | 0.320493915 |
| State_Oregon | 1999 | 0.923750232 | 0.201734357 |
| State_Oregon | 2000 | 1.256381622 | 0.23354501 |
| State_Oregon | 2001 | 0.986606336 | 0.205919864 |
| State_Oregon | 2002 | 1.960470492 | 0.289525277 |
| State_Oregon | 2003 | 1.407188986 | 0.245489198 |
| State_Oregon | 2004 | 1.407703915 | 0.241915105 |
| State_Oregon | 2005 | 1.186571787 | 0.21757885 |
| State_Oregon | 2006 | 1.096294009 | 0.204721216 |
| State_Oregon | 2007 | 1.619401829 | 0.250940279 |
| State_Oregon | 2008 | 1.345631684 | 0.226593461 |
| State_Oregon | 2009 | 1.491789923 | 0.235241629 |
| State_Oregon | 2010 | 1.464321237 | 0.233614579 |
| State_Oregon | 2011 | 1.720827321 | 0.247382685 |
| State_Oregon | 2012 | 1.527576767 | 0.228086016 |
| State_Oregon | 2013 | 1.738203929 | 0.240851043 |
| State_Oregon | 2014 | 1.367332951 | 0.211592929 |
| State_Oregon | 2015 | 1.645845057 | 0.22760685 |
| State_Oregon | 2016 | 2.024714044 | 0.251689457 |
| State_Oregon | 2017 | 1.587044923 | 0.221709735 |
| State_Oregon | 2018 | 2.195327808 | 0.254548791 |
| State_Oregon | 2019 | 1.785876155 | 0.226424457 |
| State_Oregon | 2020 | 1.530019885 | 0.209719167 |
| State_Oregon | 2021 | 2.695637094 | 0.27979624 |
| State_Oregon | 2022 | 3.065421367 | 0.287905823 |
| State_Oregon | 2023 | 2.761653402 | 0.269697486 |
| State_Oregon | 2024 | 2.332353748 | 0.246536187 |
| State_Pennsylvania | 1999 | 1.19980766 | 0.113153059 |
| State_Pennsylvania | 2000 | 1.274888304 | 0.114802713 |
| State_Pennsylvania | 2001 | 1.264244328 | 0.112837407 |
| State_Pennsylvania | 2002 | 1.487534844 | 0.124254136 |
| State_Pennsylvania | 2003 | 1.281526706 | 0.116001966 |
| State_Pennsylvania | 2004 | 1.386739879 | 0.120206506 |
| State_Pennsylvania | 2005 | 1.359707788 | 0.116502604 |
| State_Pennsylvania | 2006 | 1.447858 | 0.119701483 |
| State_Pennsylvania | 2007 | 1.42665556 | 0.118729845 |
| State_Pennsylvania | 2008 | 1.313041982 | 0.113387382 |
| State_Pennsylvania | 2009 | 1.302654003 | 0.112398623 |
| State_Pennsylvania | 2010 | 1.281799198 | 0.112103703 |
| State_Pennsylvania | 2011 | 1.443894038 | 0.118080134 |
| State_Pennsylvania | 2012 | 1.217288637 | 0.106369386 |
| State_Pennsylvania | 2013 | 1.352300268 | 0.11150053 |
| State_Pennsylvania | 2014 | 1.228612197 | 0.107494584 |
| State_Pennsylvania | 2015 | 1.230409155 | 0.105423623 |
| State_Pennsylvania | 2016 | 1.261961749 | 0.109344113 |
| State_Pennsylvania | 2017 | 1.198646208 | 0.103014426 |
| State_Pennsylvania | 2018 | 1.087875849 | 0.097587211 |
| State_Pennsylvania | 2019 | 1.289661136 | 0.106633643 |
| State_Pennsylvania | 2020 | 1.626982663 | 0.118788638 |
| State_Pennsylvania | 2021 | 1.565865909 | 0.116636437 |
| State_Pennsylvania | 2022 | 1.550848908 | 0.112314692 |
| State_Pennsylvania | 2023 | 1.517025224 | 0.111627121 |
| State_Pennsylvania | 2024 | 1.350351426 | 0.105816514 |
| State_Rhode Island | 1999 | NA | 0.462001803 |
| State_Rhode Island | 2000 | NA | 0.42828284 |
| State_Rhode Island | 2001 | NA | 0.394228069 |
| State_Rhode Island | 2002 | NA | 0.482477205 |
| State_Rhode Island | 2003 | NA | 0.368430827 |
| State_Rhode Island | 2004 | NA | 0.392788794 |
| State_Rhode Island | 2005 | 2.636302563 | 0.580955772 |
| State_Rhode Island | 2006 | NA | 0.574999927 |
| State_Rhode Island | 2007 | NA | 0.440772284 |
| State_Rhode Island | 2008 | NA | NA |
| State_Rhode Island | 2009 | NA | NA |
| State_Rhode Island | 2010 | NA | NA |
| State_Rhode Island | 2011 | NA | NA |
| State_Rhode Island | 2012 | NA | 0.473333792 |
| State_Rhode Island | 2013 | NA | 0.41041313 |
| State_Rhode Island | 2014 | NA | 0.418314036 |
| State_Rhode Island | 2015 | 2.471742822 | 0.55322548 |
| State_Rhode Island | 2016 | NA | 0.385195689 |
| State_Rhode Island | 2017 | NA | 0.392081612 |
| State_Rhode Island | 2018 | NA | 0.329579196 |
| State_Rhode Island | 2019 | NA | 0.325167957 |
| State_Rhode Island | 2020 | 2.476400267 | 0.512617301 |
| State_Rhode Island | 2021 | 1.838357553 | 0.439791657 |
| State_Rhode Island | 2022 | 1.90657757 | 0.444146001 |
| State_Rhode Island | 2023 | 2.503486908 | 0.503009134 |
| State_Rhode Island | 2024 | 1.647225133 | 0.399550117 |
| State_South Carolina | 1999 | NA | 0.167024674 |
| State_South Carolina | 2000 | NA | 0.173733796 |
| State_South Carolina | 2001 | 1.03848586 | 0.208835164 |
| State_South Carolina | 2002 | NA | 0.158170408 |
| State_South Carolina | 2003 | NA | 0.168975677 |
| State_South Carolina | 2004 | NA | 0.160920396 |
| State_South Carolina | 2005 | 1.155727409 | 0.211779001 |
| State_South Carolina | 2006 | NA | 0.127292152 |
| State_South Carolina | 2007 | 0.925100502 | 0.179316133 |
| State_South Carolina | 2008 | 1.153582227 | 0.197060175 |
| State_South Carolina | 2009 | 0.785417677 | 0.158970546 |
| State_South Carolina | 2010 | 0.760149658 | 0.15369862 |
| State_South Carolina | 2011 | 0.881602602 | 0.16870303 |
| State_South Carolina | 2012 | 1.066402928 | 0.176212706 |
| State_South Carolina | 2013 | 1.225439556 | 0.189311203 |
| State_South Carolina | 2014 | 1.004572763 | 0.167854754 |
| State_South Carolina | 2015 | 1.091912342 | 0.17149192 |
| State_South Carolina | 2016 | 1.01700192 | 0.167190742 |
| State_South Carolina | 2017 | 1.092713203 | 0.170779223 |
| State_South Carolina | 2018 | 1.153637283 | 0.166972326 |
| State_South Carolina | 2019 | 1.193095667 | 0.171262774 |
| State_South Carolina | 2020 | 0.918977683 | 0.149529687 |
| State_South Carolina | 2021 | 1.822310982 | 0.206336542 |
| State_South Carolina | 2022 | 2.468827435 | 0.23183096 |
| State_South Carolina | 2023 | 2.515791944 | 0.240993867 |
| State_South Carolina | 2024 | 2.686096018 | 0.237627426 |
| State_South Dakota | 1999 | NA | NA |
| State_South Dakota | 2000 | NA | NA |
| State_South Dakota | 2001 | NA | NA |
| State_South Dakota | 2002 | NA | 0.57604761 |
| State_South Dakota | 2003 | NA | NA |
| State_South Dakota | 2004 | NA | 0.5604883 |
| State_South Dakota | 2005 | NA | NA |
| State_South Dakota | 2006 | NA | NA |
| State_South Dakota | 2007 | NA | NA |
| State_South Dakota | 2008 | NA | NA |
| State_South Dakota | 2009 | NA | NA |
| State_South Dakota | 2010 | NA | NA |
| State_South Dakota | 2011 | NA | 0.558129139 |
| State_South Dakota | 2012 | NA | NA |
| State_South Dakota | 2013 | NA | 0.557756282 |
| State_South Dakota | 2014 | NA | 0.515300936 |
| State_South Dakota | 2015 | NA | 0.509370272 |
| State_South Dakota | 2016 | NA | 0.578704238 |
| State_South Dakota | 2017 | NA | 0.415269852 |
| State_South Dakota | 2018 | NA | 0.553343081 |
| State_South Dakota | 2019 | NA | 0.494089276 |
| State_South Dakota | 2020 | NA | 0.590840679 |
| State_South Dakota | 2021 | 2.665324743 | 0.62679955 |
| State_South Dakota | 2022 | 1.579313718 | 0.464702126 |
| State_South Dakota | 2023 | 1.551636668 | 0.464197378 |
| State_South Dakota | 2024 | 1.702460754 | 0.462913589 |
| State_Tennessee | 1999 | 0.925975189 | 0.16160257 |
| State_Tennessee | 2000 | 0.666893736 | 0.136246868 |
| State_Tennessee | 2001 | 0.908192007 | 0.158384357 |
| State_Tennessee | 2002 | 1.09111534 | 0.172865877 |
| State_Tennessee | 2003 | 0.986867833 | 0.164729554 |
| State_Tennessee | 2004 | 1.054446982 | 0.165526039 |
| State_Tennessee | 2005 | 1.298622497 | 0.18423336 |
| State_Tennessee | 2006 | 1.104518059 | 0.167409461 |
| State_Tennessee | 2007 | 1.477711785 | 0.191951069 |
| State_Tennessee | 2008 | 1.167741307 | 0.167993146 |
| State_Tennessee | 2009 | 1.229311886 | 0.170246442 |
| State_Tennessee | 2010 | 1.36117927 | 0.180033607 |
| State_Tennessee | 2011 | 1.452518541 | 0.182981932 |
| State_Tennessee | 2012 | 1.342125537 | 0.170777223 |
| State_Tennessee | 2013 | 1.373993243 | 0.16984088 |
| State_Tennessee | 2014 | 1.280402542 | 0.165442054 |
| State_Tennessee | 2015 | 0.95597998 | 0.138382487 |
| State_Tennessee | 2016 | 0.990831647 | 0.14168314 |
| State_Tennessee | 2017 | 1.237199917 | 0.154386617 |
| State_Tennessee | 2018 | 1.344570903 | 0.15795002 |
| State_Tennessee | 2019 | 1.540528795 | 0.167566078 |
| State_Tennessee | 2020 | 1.971371446 | 0.187349639 |
| State_Tennessee | 2021 | 2.051544992 | 0.196460563 |
| State_Tennessee | 2022 | 1.748137845 | 0.177161835 |
| State_Tennessee | 2023 | 1.800438086 | 0.177732606 |
| State_Tennessee | 2024 | 1.963022125 | 0.183893445 |
| State_Texas | 1999 | 0.845964001 | 0.090340621 |
| State_Texas | 2000 | 0.996990526 | 0.097108248 |
| State_Texas | 2001 | 1.162858689 | 0.103505335 |
| State_Texas | 2002 | 1.171597856 | 0.102837681 |
| State_Texas | 2003 | 1.41166644 | 0.112154665 |
| State_Texas | 2004 | 1.164202872 | 0.100481733 |
| State_Texas | 2005 | 1.205991276 | 0.101466638 |
| State_Texas | 2006 | 1.168374337 | 0.098167787 |
| State_Texas | 2007 | 1.223959963 | 0.099568365 |
| State_Texas | 2008 | 1.245814261 | 0.098534198 |
| State_Texas | 2009 | 1.442496786 | 0.104476078 |
| State_Texas | 2010 | 1.374794076 | 0.100137415 |
| State_Texas | 2011 | 1.552687487 | 0.105886056 |
| State_Texas | 2012 | 1.246588839 | 0.092728787 |
| State_Texas | 2013 | 1.55076493 | 0.102321555 |
| State_Texas | 2014 | 1.536534182 | 0.100203345 |
| State_Texas | 2015 | 1.565304283 | 0.099326644 |
| State_Texas | 2016 | 1.420291276 | 0.093251214 |
| State_Texas | 2017 | 1.377971774 | 0.090944597 |
| State_Texas | 2018 | 1.393066717 | 0.088847567 |
| State_Texas | 2019 | 1.457225205 | 0.08966553 |
| State_Texas | 2020 | 1.683626145 | 0.096248217 |
| State_Texas | 2021 | 1.796176755 | 0.100622302 |
| State_Texas | 2022 | 2.147203314 | 0.107361797 |
| State_Texas | 2023 | 1.846385109 | 0.098445312 |
| State_Texas | 2024 | 1.995122584 | 0.099312638 |
| State_Utah | 1999 | NA | NA |
| State_Utah | 2000 | NA | NA |
| State_Utah | 2001 | NA | NA |
| State_Utah | 2002 | NA | NA |
| State_Utah | 2003 | NA | 0.374412126 |
| State_Utah | 2004 | NA | NA |
| State_Utah | 2005 | NA | NA |
| State_Utah | 2006 | NA | 0.294815286 |
| State_Utah | 2007 | NA | NA |
| State_Utah | 2008 | NA | NA |
| State_Utah | 2009 | NA | NA |
| State_Utah | 2010 | NA | 0.277983751 |
| State_Utah | 2011 | NA | 0.318422995 |
| State_Utah | 2012 | NA | NA |
| State_Utah | 2013 | NA | NA |
| State_Utah | 2014 | NA | 0.236678755 |
| State_Utah | 2015 | NA | 0.250317089 |
| State_Utah | 2016 | NA | 0.253655881 |
| State_Utah | 2017 | NA | 0.235884271 |
| State_Utah | 2018 | 1.588358885 | 0.309352822 |
| State_Utah | 2019 | 1.193586252 | 0.268685159 |
| State_Utah | 2020 | 1.71284633 | 0.311288776 |
| State_Utah | 2021 | 1.034381085 | 0.244023571 |
| State_Utah | 2022 | 0.920141214 | 0.221632282 |
| State_Utah | 2023 | 1.029811083 | 0.237464861 |
| State_Utah | 2024 | 1.144122745 | 0.243007262 |
| State_Vermont | 1999 | NA | NA |
| State_Vermont | 2000 | NA | NA |
| State_Vermont | 2001 | NA | NA |
| State_Vermont | 2002 | NA | NA |
| State_Vermont | 2003 | NA | NA |
| State_Vermont | 2004 | NA | NA |
| State_Vermont | 2005 | NA | 0.769750507 |
| State_Vermont | 2006 | NA | 0.799404648 |
| State_Vermont | 2007 | NA | 0.798903351 |
| State_Vermont | 2008 | NA | NA |
| State_Vermont | 2009 | NA | NA |
| State_Vermont | 2010 | NA | 0.711936811 |
| State_Vermont | 2011 | NA | 0.733666408 |
| State_Vermont | 2012 | NA | NA |
| State_Vermont | 2013 | NA | NA |
| State_Vermont | 2014 | NA | 0.713394275 |
| State_Vermont | 2015 | NA | 0.66130156 |
| State_Vermont | 2016 | NA | NA |
| State_Vermont | 2017 | NA | 0.682068967 |
| State_Vermont | 2018 | NA | NA |
| State_Vermont | 2019 | NA | NA |
| State_Vermont | 2020 | NA | NA |
| State_Vermont | 2021 | 2.184706425 | 0.611650895 |
| State_Vermont | 2022 | 1.841364607 | 0.53516231 |
| State_Vermont | 2023 | 2.587360666 | 0.635077866 |
| State_Vermont | 2024 | 1.455256689 | 0.464374032 |
| State_Virginia | 1999 | 0.876644683 | 0.148647106 |
| State_Virginia | 2000 | 0.904816716 | 0.151062935 |
| State_Virginia | 2001 | 0.491718627 | 0.110179931 |
| State_Virginia | 2002 | 0.779635188 | 0.134494522 |
| State_Virginia | 2003 | 0.750953549 | 0.131485228 |
| State_Virginia | 2004 | 0.904579286 | 0.143986073 |
| State_Virginia | 2005 | 0.891038319 | 0.140053544 |
| State_Virginia | 2006 | 0.945000408 | 0.146173199 |
| State_Virginia | 2007 | 0.933225288 | 0.141531099 |
| State_Virginia | 2008 | 0.825529126 | 0.131476969 |
| State_Virginia | 2009 | 0.802457049 | 0.128072893 |
| State_Virginia | 2010 | 0.740934176 | 0.120097718 |
| State_Virginia | 2011 | 1.063088516 | 0.144958681 |
| State_Virginia | 2012 | 0.989564996 | 0.137114569 |
| State_Virginia | 2013 | 0.838868151 | 0.125364742 |
| State_Virginia | 2014 | 0.778107889 | 0.12039834 |
| State_Virginia | 2015 | 0.782743786 | 0.113728912 |
| State_Virginia | 2016 | 0.904190233 | 0.124556171 |
| State_Virginia | 2017 | 0.848960774 | 0.119626786 |
| State_Virginia | 2018 | 0.991591893 | 0.127594774 |
| State_Virginia | 2019 | 1.026609469 | 0.125980962 |
| State_Virginia | 2020 | 1.373934135 | 0.145814967 |
| State_Virginia | 2021 | 1.235187626 | 0.13719943 |
| State_Virginia | 2022 | 1.620755601 | 0.153399701 |
| State_Virginia | 2023 | 1.515749725 | 0.148024069 |
| State_Virginia | 2024 | 1.604845226 | 0.150110851 |
| State_Washington | 1999 | 0.983382635 | 0.169158914 |
| State_Washington | 2000 | 0.783550213 | 0.150882215 |
| State_Washington | 2001 | 1.193741949 | 0.182481003 |
| State_Washington | 2002 | 1.074901932 | 0.172749098 |
| State_Washington | 2003 | 0.981155001 | 0.163889385 |
| State_Washington | 2004 | 1.076005489 | 0.17057527 |
| State_Washington | 2005 | 1.161552752 | 0.174386177 |
| State_Washington | 2006 | 1.303349703 | 0.18041967 |
| State_Washington | 2007 | 1.278675648 | 0.178566192 |
| State_Washington | 2008 | 1.540944582 | 0.19489292 |
| State_Washington | 2009 | 1.29224443 | 0.177318644 |
| State_Washington | 2010 | 1.547560283 | 0.190709995 |
| State_Washington | 2011 | 1.597132388 | 0.192413687 |
| State_Washington | 2012 | 1.280827805 | 0.167714212 |
| State_Washington | 2013 | 1.416255756 | 0.173270087 |
| State_Washington | 2014 | 1.36182003 | 0.169026912 |
| State_Washington | 2015 | 1.284413281 | 0.162659353 |
| State_Washington | 2016 | 1.490908413 | 0.169738116 |
| State_Washington | 2017 | 1.393617297 | 0.162875953 |
| State_Washington | 2018 | 1.271348971 | 0.151160848 |
| State_Washington | 2019 | 1.502570459 | 0.1631776 |
| State_Washington | 2020 | 1.89037764 | 0.179271984 |
| State_Washington | 2021 | 1.882904401 | 0.183652627 |
| State_Washington | 2022 | 1.657396184 | 0.16376127 |
| State_Washington | 2023 | 1.766736282 | 0.171590658 |
| State_Washington | 2024 | 2.11902293 | 0.184872572 |
| State_West Virginia | 1999 | 1.504471467 | 0.328543617 |
| State_West Virginia | 2000 | 1.613407584 | 0.345296659 |
| State_West Virginia | 2001 | NA | 0.312916165 |
| State_West Virginia | 2002 | 1.762781799 | 0.352787423 |
| State_West Virginia | 2003 | 1.390879346 | 0.311648125 |
| State_West Virginia | 2004 | 2.168731381 | 0.390242313 |
| State_West Virginia | 2005 | 1.483399303 | 0.324367222 |
| State_West Virginia | 2006 | 1.649163748 | 0.337351027 |
| State_West Virginia | 2007 | 1.671065004 | 0.341460222 |
| State_West Virginia | 2008 | NA | 0.29362806 |
| State_West Virginia | 2009 | 1.401666355 | 0.306699239 |
| State_West Virginia | 2010 | 2.277262455 | 0.38687291 |
| State_West Virginia | 2011 | 2.086524828 | 0.366028522 |
| State_West Virginia | 2012 | 2.12415281 | 0.360548665 |
| State_West Virginia | 2013 | 1.553487175 | 0.315195001 |
| State_West Virginia | 2014 | 1.735038224 | 0.33917533 |
| State_West Virginia | 2015 | 1.849234032 | 0.341332713 |
| State_West Virginia | 2016 | 1.969724368 | 0.341964246 |
| State_West Virginia | 2017 | 1.170119422 | 0.263963584 |
| State_West Virginia | 2018 | NA | 0.260681615 |
| State_West Virginia | 2019 | 1.185706488 | 0.262580847 |
| State_West Virginia | 2020 | 1.497032336 | 0.295141782 |
| State_West Virginia | 2021 | 1.699968059 | 0.317394562 |
| State_West Virginia | 2022 | 2.444685707 | 0.386739646 |
| State_West Virginia | 2023 | 1.901252728 | 0.323810434 |
| State_West Virginia | 2024 | 2.125120246 | 0.341672959 |
| State_Wisconsin | 1999 | 0.643650452 | 0.1342435 |
| State_Wisconsin | 2000 | 0.834440128 | 0.152593354 |
| State_Wisconsin | 2001 | 1.287151528 | 0.188159626 |
| State_Wisconsin | 2002 | 1.052646261 | 0.168910372 |
| State_Wisconsin | 2003 | 0.94262291 | 0.157558062 |
| State_Wisconsin | 2004 | 1.418473518 | 0.191927007 |
| State_Wisconsin | 2005 | 1.167309559 | 0.174784546 |
| State_Wisconsin | 2006 | 1.383919548 | 0.189146816 |
| State_Wisconsin | 2007 | 0.974149453 | 0.154972742 |
| State_Wisconsin | 2008 | 1.284858082 | 0.17758948 |
| State_Wisconsin | 2009 | 1.132338373 | 0.168070365 |
| State_Wisconsin | 2010 | 1.111177452 | 0.165003898 |
| State_Wisconsin | 2011 | 1.497648112 | 0.190386541 |
| State_Wisconsin | 2012 | 0.694864149 | 0.123903771 |
| State_Wisconsin | 2013 | 1.276079252 | 0.172080285 |
| State_Wisconsin | 2014 | 1.557885483 | 0.187087233 |
| State_Wisconsin | 2015 | 1.349680927 | 0.173849529 |
| State_Wisconsin | 2016 | 1.327219293 | 0.168562579 |
| State_Wisconsin | 2017 | 1.572096909 | 0.183837867 |
| State_Wisconsin | 2018 | 1.577998464 | 0.186971974 |
| State_Wisconsin | 2019 | 1.536334769 | 0.175646443 |
| State_Wisconsin | 2020 | 1.885416867 | 0.195324337 |
| State_Wisconsin | 2021 | 2.069948861 | 0.208140636 |
| State_Wisconsin | 2022 | 1.984721563 | 0.198344061 |
| State_Wisconsin | 2023 | 1.926711671 | 0.194565179 |
| State_Wisconsin | 2024 | 2.284027253 | 0.207428651 |
| State_Wyoming | 1999 | NA | NA |
| State_Wyoming | 2000 | NA | NA |
| State_Wyoming | 2001 | NA | NA |
| State_Wyoming | 2002 | NA | NA |
| State_Wyoming | 2003 | NA | NA |
| State_Wyoming | 2004 | NA | NA |
| State_Wyoming | 2005 | NA | NA |
| State_Wyoming | 2006 | NA | NA |
| State_Wyoming | 2007 | NA | NA |
| State_Wyoming | 2008 | NA | NA |
| State_Wyoming | 2009 | NA | NA |
| State_Wyoming | 2010 | NA | NA |
| State_Wyoming | 2011 | NA | NA |
| State_Wyoming | 2012 | NA | NA |
| State_Wyoming | 2013 | NA | NA |
| State_Wyoming | 2014 | NA | NA |
| State_Wyoming | 2015 | NA | NA |
| State_Wyoming | 2016 | NA | NA |
| State_Wyoming | 2017 | NA | 0.806781546 |
| State_Wyoming | 2018 | NA | 0.63705309 |
| State_Wyoming | 2019 | NA | NA |
| State_Wyoming | 2020 | NA | NA |
| State_Wyoming | 2021 | 3.081237668 | 0.787009198 |
| State_Wyoming | 2022 | NA | NA |
| State_Wyoming | 2023 | NA | NA |
| State_Wyoming | 2024 | 2.433851964 | 0.714761889 |
| Urbanization_Metropolitan | 1999 | 0.875757127 | 0.025072497 |
| Urbanization_Metropolitan | 2000 | 0.911925382 | 0.025066888 |
| Urbanization_Metropolitan | 2001 | 0.939662377 | 0.0251375 |
| Urbanization_Metropolitan | 2002 | 1.010902411 | 0.025866455 |
| Urbanization_Metropolitan | 2003 | 0.984803002 | 0.025381393 |
| Urbanization_Metropolitan | 2004 | 1.014822633 | 0.02567869 |
| Urbanization_Metropolitan | 2005 | 1.03339104 | 0.025728941 |
| Urbanization_Metropolitan | 2006 | 1.089740346 | 0.026073435 |
| Urbanization_Metropolitan | 2007 | 1.087032201 | 0.025995621 |
| Urbanization_Metropolitan | 2008 | 1.076772107 | 0.025429788 |
| Urbanization_Metropolitan | 2009 | 1.059723683 | 0.025075199 |
| Urbanization_Metropolitan | 2010 | 1.092043959 | 0.025191378 |
| Urbanization_Metropolitan | 2011 | 1.138425008 | 0.025504641 |
| Urbanization_Metropolitan | 2012 | 1.124149012 | 0.024999494 |
| Urbanization_Metropolitan | 2013 | 1.115505526 | 0.024424787 |
| Urbanization_Metropolitan | 2014 | 1.114411021 | 0.024308667 |
| Urbanization_Metropolitan | 2015 | 1.068079296 | 0.023382592 |
| Urbanization_Metropolitan | 2016 | 1.109371943 | 0.023769022 |
| Urbanization_Metropolitan | 2017 | 1.121441894 | 0.023332083 |
| Urbanization_Metropolitan | 2018 | 1.132912124 | 0.023018017 |
| Urbanization_Metropolitan | 2019 | 1.132550982 | 0.022593125 |
| Urbanization_Metropolitan | 2020 | 1.340060657 | 0.024516339 |
| Urbanization_Nonmetropolitan | 1999 | 0.990481785 | 0.054140001 |
| Urbanization_Nonmetropolitan | 2000 | 1.088348271 | 0.056705718 |
| Urbanization_Nonmetropolitan | 2001 | 1.246507256 | 0.060418942 |
| Urbanization_Nonmetropolitan | 2002 | 1.271935608 | 0.061849327 |
| Urbanization_Nonmetropolitan | 2003 | 1.228487866 | 0.059278263 |
| Urbanization_Nonmetropolitan | 2004 | 1.358784323 | 0.06230833 |
| Urbanization_Nonmetropolitan | 2005 | 1.278234183 | 0.059901802 |
| Urbanization_Nonmetropolitan | 2006 | 1.31641815 | 0.060395802 |
| Urbanization_Nonmetropolitan | 2007 | 1.39150716 | 0.061973507 |
| Urbanization_Nonmetropolitan | 2008 | 1.389209429 | 0.060782097 |
| Urbanization_Nonmetropolitan | 2009 | 1.29864744 | 0.058694665 |
| Urbanization_Nonmetropolitan | 2010 | 1.468197277 | 0.062474857 |
| Urbanization_Nonmetropolitan | 2011 | 1.471002017 | 0.061808519 |
| Urbanization_Nonmetropolitan | 2012 | 1.449474017 | 0.060752729 |
| Urbanization_Nonmetropolitan | 2013 | 1.465338275 | 0.060733995 |
| Urbanization_Nonmetropolitan | 2014 | 1.463468047 | 0.060865599 |
| Urbanization_Nonmetropolitan | 2015 | 1.524658219 | 0.06103842 |
| Urbanization_Nonmetropolitan | 2016 | 1.559883787 | 0.062315155 |
| Urbanization_Nonmetropolitan | 2017 | 1.58086788 | 0.061878636 |
| Urbanization_Nonmetropolitan | 2018 | 1.524177097 | 0.060031815 |
| Urbanization_Nonmetropolitan | 2019 | 1.679442034 | 0.062415326 |
| Urbanization_Nonmetropolitan | 2020 | 1.924430032 | 0.067551893 |
| Age Groups_25-34 years | 1999 | NA | NA |
| Age Groups_25-34 years | 2000 | NA | 0 |
| Age Groups_25-34 years | 2001 | NA | 0 |
| Age Groups_25-34 years | 2002 | NA | 0 |
| Age Groups_25-34 years | 2003 | NA | NA |
| Age Groups_25-34 years | 2004 | NA | 0 |
| Age Groups_25-34 years | 2005 | NA | 0 |
| Age Groups_25-34 years | 2006 | NA | NA |
| Age Groups_25-34 years | 2007 | NA | NA |
| Age Groups_25-34 years | 2008 | NA | 0 |
| Age Groups_25-34 years | 2009 | NA | 0 |
| Age Groups_25-34 years | 2010 | NA | NA |
| Age Groups_25-34 years | 2011 | NA | 0 |
| Age Groups_25-34 years | 2012 | NA | 0 |
| Age Groups_25-34 years | 2013 | NA | 0 |
| Age Groups_25-34 years | 2014 | NA | 0 |
| Age Groups_25-34 years | 2015 | NA | NA |
| Age Groups_25-34 years | 2016 | NA | 0 |
| Age Groups_25-34 years | 2017 | NA | 0 |
| Age Groups_25-34 years | 2018 | NA | NA |
| Age Groups_25-34 years | 2019 | NA | 0 |
| Age Groups_25-34 years | 2020 | NA | 0 |
| Age Groups_25-34 years | 2021 | NA | NA |
| Age Groups_25-34 years | 2022 | 0 | 0 |
| Age Groups_25-34 years | 2023 | NA | NA |
| Age Groups_25-34 years | 2024 | NA | NA |
| Age Groups_35-44 years | 1999 | NA | NA |
| Age Groups_35-44 years | 2000 | NA | NA |
| Age Groups_35-44 years | 2001 | NA | NA |
| Age Groups_35-44 years | 2002 | NA | 0.007759971 |
| Age Groups_35-44 years | 2003 | NA | 0.007511458 |
| Age Groups_35-44 years | 2004 | NA | 0.007219767 |
| Age Groups_35-44 years | 2005 | NA | 0.007268678 |
| Age Groups_35-44 years | 2006 | NA | 0.007669596 |
| Age Groups_35-44 years | 2007 | NA | NA |
| Age Groups_35-44 years | 2008 | NA | NA |
| Age Groups_35-44 years | 2009 | NA | NA |
| Age Groups_35-44 years | 2010 | NA | NA |
| Age Groups_35-44 years | 2011 | NA | NA |
| Age Groups_35-44 years | 2012 | NA | 0.008185878 |
| Age Groups_35-44 years | 2013 | NA | 0.008563341 |
| Age Groups_35-44 years | 2014 | NA | NA |
| Age Groups_35-44 years | 2015 | NA | NA |
| Age Groups_35-44 years | 2016 | NA | 0.008559645 |
| Age Groups_35-44 years | 2017 | NA | 0.008113993 |
| Age Groups_35-44 years | 2018 | NA | NA |
| Age Groups_35-44 years | 2019 | NA | NA |
| Age Groups_35-44 years | 2020 | NA | 0.009191584 |
| Age Groups_35-44 years | 2021 | NA | NA |
| Age Groups_35-44 years | 2022 | 0.034328584 | 0.008863602 |
| Age Groups_35-44 years | 2023 | 0.031538142 | 0.008428923 |
| Age Groups_35-44 years | 2024 | 0.02635091 | 0.007606852 |
| Age Groups_45-54 years | 1999 | 0.144896556 | 0.019903073 |
| Age Groups_45-54 years | 2000 | 0.114125099 | 0.017403914 |
| Age Groups_45-54 years | 2001 | 0.152337358 | 0.019666668 |
| Age Groups_45-54 years | 2002 | 0.140027326 | 0.018711939 |
| Age Groups_45-54 years | 2003 | 0.151886501 | 0.019289605 |
| Age Groups_45-54 years | 2004 | 0.139322838 | 0.018293985 |
| Age Groups_45-54 years | 2005 | 0.155309086 | 0.019117227 |
| Age Groups_45-54 years | 2006 | 0.157094096 | 0.019050457 |
| Age Groups_45-54 years | 2007 | 0.163860036 | 0.01931109 |
| Age Groups_45-54 years | 2008 | 0.155194121 | 0.018683177 |
| Age Groups_45-54 years | 2009 | 0.140414729 | 0.017690593 |
| Age Groups_45-54 years | 2010 | 0.139979109 | 0.01763571 |
| Age Groups_45-54 years | 2011 | 0.156535807 | 0.018709607 |
| Age Groups_45-54 years | 2012 | 0.160384062 | 0.019034086 |
| Age Groups_45-54 years | 2013 | 0.143942318 | 0.018135027 |
| Age Groups_45-54 years | 2014 | 0.172577043 | 0.019927481 |
| Age Groups_45-54 years | 2015 | 0.141242411 | 0.018084238 |
| Age Groups_45-54 years | 2016 | 0.161265145 | 0.019414042 |
| Age Groups_45-54 years | 2017 | 0.160472158 | 0.019460108 |
| Age Groups_45-54 years | 2018 | 0.165739092 | 0.019952642 |
| Age Groups_45-54 years | 2019 | 0.15412881 | 0.019418405 |
| Age Groups_45-54 years | 2020 | 0.210572561 | 0.022839801 |
| Age Groups_45-54 years | 2021 | 0.191700659 | 0.021705825 |
| Age Groups_45-54 years | 2022 | 0.257224261 | 0.025222914 |
| Age Groups_45-54 years | 2023 | 0.227195381 | 0.023686756 |
| Age Groups_45-54 years | 2024 | 0.232955298 | 0.023900709 |
| Age Groups_55-64 years | 1999 | 0.681301299 | 0.053528085 |
| Age Groups_55-64 years | 2000 | 0.803306029 | 0.057525939 |
| Age Groups_55-64 years | 2001 | 0.804611139 | 0.056612242 |
| Age Groups_55-64 years | 2002 | 0.756459905 | 0.053224333 |
| Age Groups_55-64 years | 2003 | 0.810455374 | 0.053791813 |
| Age Groups_55-64 years | 2004 | 0.815552024 | 0.052753675 |
| Age Groups_55-64 years | 2005 | 0.750616068 | 0.049494158 |
| Age Groups_55-64 years | 2006 | 0.742246042 | 0.048214062 |
| Age Groups_55-64 years | 2007 | 0.751620194 | 0.047631994 |
| Age Groups_55-64 years | 2008 | 0.802176698 | 0.048461267 |
| Age Groups_55-64 years | 2009 | 0.731522697 | 0.045454609 |
| Age Groups_55-64 years | 2010 | 0.857940205 | 0.048493648 |
| Age Groups_55-64 years | 2011 | 0.859121426 | 0.047509523 |
| Age Groups_55-64 years | 2012 | 0.818945591 | 0.046069289 |
| Age Groups_55-64 years | 2013 | 0.864778392 | 0.046899193 |
| Age Groups_55-64 years | 2014 | 0.788470741 | 0.044354945 |
| Age Groups_55-64 years | 2015 | 0.785266944 | 0.043829327 |
| Age Groups_55-64 years | 2016 | 0.757299061 | 0.042736858 |
| Age Groups_55-64 years | 2017 | 0.819132302 | 0.044164654 |
| Age Groups_55-64 years | 2018 | 0.806668408 | 0.043683543 |
| Age Groups_55-64 years | 2019 | 0.805681477 | 0.043566265 |
| Age Groups_55-64 years | 2020 | 0.995196714 | 0.048445418 |
| Age Groups_55-64 years | 2021 | 0.967220477 | 0.047536293 |
| Age Groups_55-64 years | 2022 | 1.014602747 | 0.049100068 |
| Age Groups_55-64 years | 2023 | 0.99153229 | 0.048672406 |
| Age Groups_55-64 years | 2024 | 1.020120986 | 0.049483136 |
| Age Groups_65-74 years | 1999 | 2.334557383 | 0.112582354 |
| Age Groups_65-74 years | 2000 | 2.544724899 | 0.11762995 |
| Age Groups_65-74 years | 2001 | 2.616380095 | 0.119296664 |
| Age Groups_65-74 years | 2002 | 3.018184972 | 0.128114817 |
| Age Groups_65-74 years | 2003 | 2.680948483 | 0.12037814 |
| Age Groups_65-74 years | 2004 | 2.860581524 | 0.123789469 |
| Age Groups_65-74 years | 2005 | 2.90757764 | 0.12409239 |
| Age Groups_65-74 years | 2006 | 2.989112081 | 0.124763128 |
| Age Groups_65-74 years | 2007 | 3.040805632 | 0.12424395 |
| Age Groups_65-74 years | 2008 | 2.847992 | 0.117851 |
| Age Groups_65-74 years | 2009 | 2.835196 | 0.115554 |
| Age Groups_65-74 years | 2010 | 2.735634 | 0.112244 |
| Age Groups_65-74 years | 2011 | 2.997989 | 0.115478 |
| Age Groups_65-74 years | 2012 | 2.951797 | 0.110935 |
| Age Groups_65-74 years | 2013 | 2.851278 | 0.106335 |
| Age Groups_65-74 years | 2014 | 2.848669 | 0.10388 |
| Age Groups_65-74 years | 2015 | 2.74042 | 0.099734 |
| Age Groups_65-74 years | 2016 | 2.724384 | 0.097549 |
| Age Groups_65-74 years | 2017 | 2.900607 | 0.098852 |
| Age Groups_65-74 years | 2018 | 2.826942 | 0.096286 |
| Age Groups_65-74 years | 2019 | 3.023813 | 0.098002 |
| Age Groups_65-74 years | 2020 | 3.348756 | 0.101431 |
| Age Groups_65-74 years | 2021 | 3.522829 | 0.102294 |
| Age Groups_65-74 years | 2022 | 3.521915 | 0.102095 |
| Age Groups_65-74 years | 2023 | 3.278047 | 0.097215 |
| Age Groups_65-74 years | 2024 | 3.190862 | 0.09488 |
| Age Groups_75-84 years | 1999 | 5.235211 | 0.20694 |
| Age Groups_75-84 years | 2000 | 5.355476 | 0.208146 |
| Age Groups_75-84 years | 2001 | 5.947457 | 0.217315 |
| Age Groups_75-84 years | 2002 | 6.134025 | 0.219212 |
| Age Groups_75-84 years | 2003 | 6.23428 | 0.219866 |
| Age Groups_75-84 years | 2004 | 6.281802 | 0.219907 |
| Age Groups_75-84 years | 2005 | 6.546944 | 0.22377 |
| Age Groups_75-84 years | 2006 | 6.811682 | 0.228072 |
| Age Groups_75-84 years | 2007 | 6.548264 | 0.223684 |
| Age Groups_75-84 years | 2008 | 6.691597 | 0.226217 |
| Age Groups_75-84 years | 2009 | 6.734356 | 0.227403 |
| Age Groups_75-84 years | 2010 | 6.974898 | 0.231089 |
| Age Groups_75-84 years | 2011 | 7.2105 | 0.23394 |
| Age Groups_75-84 years | 2012 | 6.426757 | 0.220048 |
| Age Groups_75-84 years | 2013 | 7.050152 | 0.228978 |
| Age Groups_75-84 years | 2014 | 6.965005 | 0.225619 |
| Age Groups_75-84 years | 2015 | 6.973985 | 0.223806 |
| Age Groups_75-84 years | 2016 | 7.15915 | 0.224272 |
| Age Groups_75-84 years | 2017 | 6.833689 | 0.215562 |
| Age Groups_75-84 years | 2018 | 7.197435 | 0.216226 |
| Age Groups_75-84 years | 2019 | 7.094609 | 0.210772 |
| Age Groups_75-84 years | 2020 | 8.193758 | 0.223171 |
| Age Groups_75-84 years | 2020 | 8.193758 | 0.223171 |
| Age Groups_75-84 years | 2021 | 9.39154 | 0.24073 |
| Age Groups_75-84 years | 2022 | 9.440346 | 0.232124 |
| Age Groups_75-84 years | 2023 | 9.119072 | 0.222814 |
| Age Groups_75-84 years | 2024 | 8.984543 | 0.21576 |
| Age Groups_85+ years | 1999 | 6.596023 | 0.39848 |
| Age Groups_85+ years | 2000 | 7.689428 | 0.425878 |
| Age Groups_85+ years | 2001 | 7.814504 | 0.425683 |
| Age Groups_85+ years | 2002 | 8.308903 | 0.436104 |
| Age Groups_85+ years | 2003 | 7.993415 | 0.423056 |
| Age Groups_85+ years | 2004 | 8.777173 | 0.439408 |
| Age Groups_85+ years | 2005 | 8.032729 | 0.413707 |
| Age Groups_85+ years | 2006 | 9.597345 | 0.444112 |
| Age Groups_85+ years | 2007 | 9.842158 | 0.441926 |
| Age Groups_85+ years | 2008 | 10.39293 | 0.44724 |
| Age Groups_85+ years | 2009 | 9.371563 | 0.417857 |
| Age Groups_85+ years | 2010 | 10.66728 | 0.440661 |
| Age Groups_85+ years | 2011 | 10.1618 | 0.420859 |
| Age Groups_85+ years | 2012 | 11.78803 | 0.447467 |
| Age Groups_85+ years | 2013 | 10.97539 | 0.426249 |
| Age Groups_85+ years | 2014 | 10.67795 | 0.41627 |
| Age Groups_85+ years | 2015 | 10.75207 | 0.413541 |
| Age Groups_85+ years | 2016 | 11.25333 | 0.419971 |
| Age Groups_85+ years | 2017 | 11.70254 | 0.425336 |
| Age Groups_85+ years | 2018 | 11.46 | 0.41846 |
| Age Groups_85+ years | 2019 | 12.4452 | 0.434076 |
| Age Groups_85+ years | 2020 | 14.46289 | 0.46606 |
| Age Groups_85+ years | 2021 | 16.11512 | 0.519302 |
| Age Groups_85+ years | 2022 | 15.49523 | 0.488782 |
| Age Groups_85+ years | 2023 | 16.27124 | 0.512496 |
| Age Groups_85+ years | 2024 | 16.9227 | 0.512809 |

Supplementary table 3. Characteristics with APC, 95% CI, and Time Range.

| Metric | Measure | Start | End | APC | Lower | Upper | *P* value |
| --- | --- | --- | --- | --- | --- | --- | --- |
| Census Region | Midwest | 1999 | 2001 | 11.7164 | -3.1838 | 28.9098 | 0.119788 |
| Census Region | Midwest | 2001 | 2018 | 0.4058 | -0.0623 | 0.8761 | 0.084514 |
| Census Region | Midwest | 2018 | 2021 | 7.223 | -3.5789 | 19.2349 | 0.181889 |
| Census Region | Midwest | 2021 | 2024 | -4.0609 | -8.7817 | 0.9043 | 0.10033 |
| Census Region | Northeast | 1999 | 2006 | 2.4642 | 0.5708 | 4.3933 | 0.013962 |
| Census Region | Northeast | 2006 | 2018 | -1.0096 | -1.8716 | -0.14 | 0.025856 |
| Census Region | Northeast | 2018 | 2021 | 9.0358 | -3.0424 | 22.6186 | 0.137143 |
| Census Region | Northeast | 2021 | 2024 | -1.9536 | -7.1208 | 3.5011 | 0.449404 |
| Census Region | South | 1999 | 2002 | 8.4996 | 1.3613 | 16.1407 | 0.021983 |
| Census Region | South | 2002 | 2018 | 1.3907 | 0.9292 | 1.8542 | 0.000011 |
| Census Region | South | 2018 | 2022 | 10.9145 | 6.4676 | 15.5471 | 0.000074 |
| Census Region | South | 2022 | 2024 | -0.1505 | -6.913 | 7.1034 | 0.964103 |
| Census Region | West | 1999 | 2024 | 1.678 | 1.3262 | 2.031 | 0 |
| Race | Hispanic | 1999 | 2024 | 1.0086 | 0.4854 | 1.5345 | 0.000543 |
| Race | NH Black | 1999 | 2018 | -0.4053 | -1.1896 | 0.3852 | 0.297481 |
| Race | NH Black | 2018 | 2024 | 7.8247 | 4.2449 | 11.5274 | 0.000141 |
| Race | NH Other | 1999 | 2024 | 0.8078 | -0.0354 | 1.6582 | 0.059657 |
| Race | NH White | 1999 | 2001 | 7.8551 | -0.3796 | 16.7704 | 0.060208 |
| Race | NH White | 2001 | 2011 | 1.7318 | 1.0881 | 2.3795 | 0.000073 |
| Race | NH White | 2011 | 2018 | 0.3018 | -0.7425 | 1.3571 | 0.542195 |
| Race | NH White | 2018 | 2021 | 10.3269 | 4.4255 | 16.5618 | 0.002129 |
| Race | NH White | 2021 | 2024 | -0.6634 | -3.1313 | 1.8674 | 0.574984 |
| Sex | Both | 1999 | 2001 | 8.9308 | 0.2816 | 18.3259 | 0.04377 |
| Sex | Both | 2001 | 2011 | 1.4234 | 0.761 | 2.0902 | 0.000515 |
| Sex | Both | 2011 | 2018 | -0.0632 | -1.1286 | 1.0137 | 0.899916 |
| Sex | Both | 2018 | 2021 | 9.9254 | 3.9099 | 16.2891 | 0.003244 |
| Sex | Both | 2021 | 2024 | -0.7523 | -3.2383 | 1.7977 | 0.528849 |
| Sex | Female | 1999 | 2011 | 1.0011 | 0.1532 | 1.8561 | 0.02362 |
| Sex | Female | 2011 | 2018 | -2.2806 | -4.4202 | -0.0931 | 0.042162 |
| Sex | Female | 2018 | 2021 | 10.6483 | -1.9819 | 24.9061 | 0.095435 |
| Sex | Female | 2021 | 2024 | -0.4063 | -5.6067 | 5.0805 | 0.873605 |
| Sex | Male | 1999 | 2002 | 7.2388 | 3.2521 | 11.3794 | 0.001331 |
| Sex | Male | 2002 | 2018 | 0.9197 | 0.6613 | 1.1788 | 0.000002 |
| Sex | Male | 2018 | 2021 | 9.2892 | 4.0967 | 14.7407 | 0.001452 |
| Sex | Male | 2021 | 2024 | -0.8624 | -3.029 | 1.3527 | 0.416598 |
| State | California | 1999 | 2006 | 4.1573 | 0.9897 | 7.4243 | 0.012194 |
| State | California | 2006 | 2024 | -0.1955 | -0.7883 | 0.4007 | 0.501883 |
| State | Florida | 1999 | 2008 | 4.5902 | 1.9245 | 7.3257 | 0.002116 |
| State | Florida | 2008 | 2011 | -6.6276 | -27.8425 | 20.8246 | 0.579044 |
| State | Florida | 2011 | 2020 | 7.0735 | 4.676 | 9.5258 | 0.000011 |
| State | Florida | 2020 | 2024 | -0.0899 | -5.1631 | 5.2547 | 0.971143 |
| State | Georgia | 1999 | 2018 | 0.4111 | -1.153 | 2.0001 | 0.592524 |
| State | Georgia | 2018 | 2024 | 13.3145 | 7.0629 | 19.9312 | 0.000162 |
| State | Illinois | 1999 | 2024 | -0.2046 | -0.7498 | 0.3436 | 0.447823 |
| State | Indiana | 1999 | 2024 | 2.0521 | 1.4018 | 2.7066 | 0.000001 |
| State | Maryland | 1999 | 2016 | -1.523 | -3.2823 | 0.2682 | 0.091144 |
| State | Maryland | 2016 | 2024 | 10.9925 | 6.6583 | 15.5027 | 0.000021 |
| State | Massachusetts | 1999 | 2024 | -0.8288 | -1.6418 | -0.0091 | 0.047722 |
| State | Michigan | 1999 | 2024 | 0.0975 | -0.6915 | 0.8929 | 0.801469 |
| State | Minnesota | 1999 | 2024 | 3.1222 | 2.5088 | 3.7392 | 0 |
| State | Missouri | 1999 | 2024 | 1.0777 | 0.2294 | 1.9332 | 0.014842 |
| State | New Jersey | 1999 | 2024 | 1.0721 | 0.1345 | 2.0184 | 0.026655 |
| State | New York | 1999 | 2024 | 1.9997 | 1.2631 | 2.7416 | 0.000008 |
| State | North Carolina | 1999 | 2009 | 4.7024 | 0.5749 | 8.9994 | 0.027408 |
| State | North Carolina | 2009 | 2018 | -6.3379 | -11.0828 | -1.3399 | 0.016425 |
| State | North Carolina | 2018 | 2024 | 14.6283 | 7.4694 | 22.2641 | 0.000311 |
| State | Ohio | 1999 | 2004 | 7.7242 | -0.1409 | 16.2087 | 0.054026 |
| State | Ohio | 2004 | 2024 | -1.5676 | -2.3951 | -0.733 | 0.000841 |
| State | Oregon | 1999 | 2024 | 3.4115 | 2.3246 | 4.5099 | 0.000001 |
| State | Pennsylvania | 1999 | 2006 | 1.8793 | -0.4487 | 4.2617 | 0.106588 |
| State | Pennsylvania | 2006 | 2018 | -1.5935 | -2.6849 | -0.4899 | 0.007779 |
| State | Pennsylvania | 2018 | 2021 | 12.3742 | -3.3655 | 30.6777 | 0.120158 |
| State | Pennsylvania | 2021 | 2024 | -6.1281 | -12.7286 | 0.9715 | 0.084294 |
| State | Tennessee | 1999 | 2011 | 4.6559 | 2.2724 | 7.0949 | 0.000757 |
| State | Tennessee | 2011 | 2016 | -7.5717 | -16.847 | 2.7382 | 0.133368 |
| State | Tennessee | 2016 | 2020 | 17.6873 | 1.8255 | 36.0199 | 0.029953 |
| State | Tennessee | 2020 | 2024 | -0.616 | -7.7705 | 7.0934 | 0.862418 |
| State | Texas | 1999 | 2014 | 2.7215 | 1.4768 | 3.9815 | 0.000288 |
| State | Texas | 2014 | 2018 | -3.3599 | -13.7855 | 8.3265 | 0.53302 |
| State | Texas | 2018 | 2022 | 10.9376 | 0.0174 | 23.05 | 0.049662 |
| State | Texas | 2022 | 2024 | -2.8345 | -18.9981 | 16.5544 | 0.740889 |
| State | Virginia | 1999 | 2017 | 0.579 | -0.7949 | 1.9719 | 0.39259 |
| State | Virginia | 2017 | 2024 | 9.6797 | 5.4622 | 14.0659 | 0.000076 |
| State | Washington | 1999 | 2024 | 2.5019 | 1.8214 | 3.1869 | 0 |
| State | Wisconsin | 1999 | 2024 | 3.3058 | 2.3622 | 4.258 | 0 |
| Urbanization | Metropolitan | 1999 | 2006 | 2.884 | 1.8311 | 3.9479 | 0.000037 |
| Urbanization | Metropolitan | 2006 | 2018 | 0.2167 | -0.2216 | 0.6569 | 0.307364 |
| Urbanization | Metropolitan | 2018 | 2020 | 8.3009 | 2.4606 | 14.474 | 0.008065 |
| Urbanization | Nonmetropolitan | 1999 | 2001 | 12.6936 | 0.721 | 26.0893 | 0.038647 |
| Urbanization | Nonmetropolitan | 2001 | 2018 | 1.3733 | 1.0173 | 1.7306 | 0.000001 |
| Urbanization | Nonmetropolitan | 2018 | 2020 | 9.9395 | 1.89 | 18.6248 | 0.018195 |
| Age Groups | 45-54 years | 1999 | 2017 | 0.6372 | -0.2981 | 1.5813 | 0.171828 |
| Age Groups | 45-54 years | 2017 | 2024 | 6.6408 | 3.1105 | 10.292 | 0.000695 |
| Age Groups | 55-64 years | 1999 | 2017 | 0.3296 | -0.2985 | 0.9617 | 0.288237 |
| Age Groups | 55-64 years | 2017 | 2024 | 3.7599 | 1.6053 | 5.9602 | 0.001468 |
| Age Groups | 65-74 years | 1999 | 2002 | 7.725 | 0.9437 | 14.9618 | 0.027613 |
| Age Groups | 65-74 years | 2002 | 2018 | -0.1999 | -0.6578 | 0.2602 | 0.368577 |
| Age Groups | 65-74 years | 2018 | 2021 | 8.2738 | -0.7444 | 18.1114 | 0.070338 |
| Age Groups | 65-74 years | 2021 | 2024 | -3.7635 | -7.6018 | 0.2342 | 0.062895 |
| Age Groups | 75-84 years | 1999 | 2003 | 5.3188 | 1.9215 | 8.8294 | 0.004221 |
| Age Groups | 75-84 years | 2003 | 2019 | 0.7697 | 0.3871 | 1.1536 | 0.000636 |
| Age Groups | 75-84 years | 2019 | 2022 | 10.337 | 2.5822 | 18.6779 | 0.011517 |
| Age Groups | 75-84 years | 2022 | 2024 | -4.509 | -10.6751 | 2.0829 | 0.161367 |
| Age Groups | 85+ years | 1999 | 2012 | 3.5495 | 2.462 | 4.6487 | 0.000002 |
| Age Groups | 85+ years | 2012 | 2015 | -2.3897 | -16.7985 | 14.5143 | 0.754024 |
| Age Groups | 85+ years | 2015 | 2024 | 5.7749 | 4.4443 | 7.1224 | 0 |

Supplementary table 4. Characteristics with AAPC, 95% CI, and Time Range.

| Metric | Measure | AAPC | Lower | Upper | *P* value |
| --- | --- | --- | --- | --- | --- |
| Census Region | Midwest | 1.5125 | -0.1949 | 3.249 | 0.082821 |
| Census Region | Northeast | 1.0009 | -0.5565 | 2.5827 | 0.209087 |
| Census Region | South | 3.5705 | 2.4123 | 4.7417 | 0 |
| Census Region | West | 1.678 | 1.3262 | 2.031 | 0 |
| Race | Hispanic | 1.0086 | 0.4854 | 1.5345 | 0.000543 |
| Race | NH Black | 1.5107 | 0.5502 | 2.4804 | 0.001994 |
| Race | NH Other | 0.8078 | -0.0354 | 1.6582 | 0.059657 |
| Race | NH White | 2.5065 | 1.5525 | 3.4694 | 0 |
| Sex | Both | 2.3031 | 1.3228 | 3.2928 | 0.000004 |
| Sex | Female | 1.0026 | -0.6137 | 2.6451 | 0.225501 |
| Sex | Male | 2.4153 | 1.6616 | 3.1745 | 0 |
| State | California | 1.0046 | 0.0899 | 1.9276 | 0.031276 |
| State | Florida | 3.2918 | 0.0815 | 6.6052 | 0.044377 |
| State | Georgia | 3.3672 | 1.6181 | 5.1464 | 0.000143 |
| State | Illinois | -0.2046 | -0.7498 | 0.3436 | 0.447823 |
| State | Indiana | 2.0521 | 1.4018 | 2.7066 | 0.000001 |
| State | Maryland | 2.3202 | 0.6291 | 4.0398 | 0.006988 |
| State | Massachusetts | -0.8288 | -1.6418 | -0.0091 | 0.047722 |
| State | Michigan | 0.0975 | -0.6915 | 0.8929 | 0.801469 |
| State | Minnesota | 3.1222 | 2.5088 | 3.7392 | 0 |
| State | Missouri | 1.0777 | 0.2294 | 1.9332 | 0.014842 |
| State | New Jersey | 1.0721 | 0.1345 | 2.0184 | 0.026655 |
| State | New York | 1.9997 | 1.2631 | 2.7416 | 0.000008 |
| State | North Carolina | 2.7959 | 0.0398 | 5.6279 | 0.046736 |
| State | Ohio | 0.2243 | -1.3314 | 1.8046 | 0.778895 |
| State | Oregon | 3.4115 | 2.3246 | 4.5099 | 0.000001 |
| State | Pennsylvania | 0.3921 | -1.5998 | 2.4244 | 0.701902 |
| State | Tennessee | 3.1656 | -0.1332 | 6.5734 | 0.060166 |
| State | Texas | 2.5263 | -0.2231 | 5.3515 | 0.072028 |
| State | Virginia | 3.0482 | 1.6222 | 4.4942 | 0.000024 |
| State | Washington | 2.5019 | 1.8214 | 3.1869 | 0 |
| State | Wisconsin | 3.3058 | 2.3622 | 4.258 | 0 |
| Urbanization | Metropolitan | 1.8478 | 1.2191 | 2.4803 | 0 |
| Urbanization | Nonmetropolitan | 3.1947 | 1.9548 | 4.4497 | 0 |
| Age Groups | 45-54 years | 2.2833 | 1.1732 | 3.4055 | 0.00005 |
| Age Groups | 55-64 years | 1.2785 | 0.5733 | 1.9887 | 0.000366 |
| Age Groups | 65-74 years | 1.2664 | -0.0494 | 2.5996 | 0.059306 |
| Age Groups | 75-84 years | 2.154 | 1.0547 | 3.2653 | 0.000113 |
| Age Groups | 85+ years | 3.6082 | 1.6502 | 5.6039 | 0.000271 |
